# Transcriptional insights into *Chlorella* sp. ABC-001: a comparative study of carbon fixation and lipid synthesis under different CO_2_ conditions

**DOI:** 10.1186/s13068-023-02358-4

**Published:** 2023-07-15

**Authors:** Hyun Gi Koh, Jun Muk Cho, Seungjib Jeon, Yong Keun Chang, Bongsoo Lee, Nam Kyu Kang

**Affiliations:** 1 Carl R. Woese Institute for Genomic Biology, University of Illinois at Urbana-Champaign, Urbana, IL USA; 2 Department of Chemical and Biomolecular Engineering, KAIST, 291 Daehak-ro, Yuseong-gu, Daejeon, 34141 Republic of Korea; 3 Department of Microbial Biotechnology, College of Science and Technology, Mokwon University, 88 Doanbuk-ro, Seo-gu, Daejeon, 35349 Republic of Korea; 4 Department of Chemical Engineering, College of Engineering, Kyung Hee University, Yongin, 17104 Republic of Korea

**Keywords:** Microalgae, *Chlorella*, Transcriptomic analysis, CO_2_ fixation, Lipid accumulation

## Abstract

**Background:**

Microalgae's low tolerance to high CO_2_ concentrations presents a significant challenge for its industrial application, especially when considering the utilization of industrial exhaust gas streams with high CO_2_ content—an economically and environmentally attractive option. Therefore, the objectives of this study were to investigate the metabolic changes in carbon fixation and lipid accumulation of microalgae under ambient air and high CO_2_ conditions, deepen our understanding of the molecular mechanisms driving these processes, and identify potential target genes for metabolic engineering in microalgae. To accomplish these goals, we conducted a transcriptomic analysis of the high CO_2_-tolerant strain, *Chlorella* sp. ABC-001, under two different carbon dioxide levels (ambient air and 10% CO_2_) and at various growth phases.

**Results:**

Cells cultivated with 10% CO_2_ exhibited significantly better growth and lipid accumulation rates, achieving up to 2.5-fold higher cell density and twice the lipid content by day 7. To understand the relationship between CO_2_ concentrations and phenotypes, transcriptomic analysis was conducted across different CO_2_ conditions and growth phases. According to the analysis of differentially expressed genes and gene ontology, *Chlorella* sp. ABC-001 exhibited the development of chloroplast organelles during the early exponential phase under high CO_2_ conditions, resulting in improved CO_2_ fixation and enhanced photosynthesis. Cobalamin-independent methionine synthase expression was also significantly elevated during the early growth stage, likely contributing to the methionine supply required for various metabolic activities and active proliferation. Conversely, the cells showed sustained repression of carbonic anhydrase and ferredoxin hydrogenase, involved in the carbon concentrating mechanism, throughout the cultivation period under high CO_2_ conditions. This study also delved into the transcriptomic profiles in the Calvin cycle, nitrogen reductase, and lipid synthesis. Particularly, *Chlorella* sp. ABC-001 showed high expression levels of genes involved in lipid synthesis, such as glycerol-3-phosphate dehydrogenase and phospholipid-diacylglycerol acyltransferase. These findings suggest potential targets for metabolic engineering aimed at enhancing lipid production in microalgae.

**Conclusions:**

We expect that our findings will help understand the carbon concentrating mechanism, photosynthesis, nitrogen assimilation, and lipid accumulation metabolisms of green algae according to CO_2_ concentrations. This study also provides insights into systems metabolic engineering of microalgae for improved performance in the future.

**Supplementary Information:**

The online version contains supplementary material available at 10.1186/s13068-023-02358-4.

## Background

Due to the massive use of fossil fuels and deforestation, the carbon dioxide in the atmosphere is continuously increasing, causing global warming. Hence, the demand for sustainable alternative energy is promoting research on microalgae because of their potential for CO_2_ reduction and biofuel production. Microalgae are known to have much higher photosynthetic efficiency as compared to terrestrial plants [1, 2]. Indeed, around 27,200 tons of CO_2_ are commercially converted into microalgal biomass annually [3–5]. As such, microalgae are considered to be one of the best candidates for CO_2_ sequestration [6]. However, microalgae's low tolerance to high CO_2_ concentrations poses a significant challenge to its commercialization [7]. This is especially important when considering the utilization of industrial exhaust gas streams with high CO_2_ content (> 10%), which is the most economical and environmentally attractive option [8–11].

*Chlorella* sp. is one of the most studied microalgae for lipid and CO_2_ sequestration [12, 13]. A whole genome sequence has been unveiled in several *Chlorella* sp., and diverse genetic manipulation tools, including CRISPR/Cas9 and transformation methods, have been developed [14–16]. Recently, multiple transcriptomic analyses have been carried out under diverse conditions to understand the mechanisms of CO_2_ fixation, lipid accumulation, and stress resistance in *Chlorella* sp. (Table 1) [13, 17–28]. In particular, research has been conducted on the photosynthesis of these strains under high CO_2_ concentrations, as *Chlorella* is known to have strains that have a tolerance to high CO_2_ compared to other species [13, 22, 28–30].

**Table 1:** Transcriptomic studies conducted in *Chlorella* sp.

Species	Habitat	Purpose of transcriptomics	Variables	Lipid Analysis	Carbon source	Analyzed samples	Ref.
*Chlorella pyrenoidosa* FACHB-9	Freshwater	Redirection of central metabolism when CO_2_ is deprived	Growth after CO_2_ stress	n.a	5% CO_2_ → AA	0 h, 1 h, 4 h, 24 h	[21]
*Chlorella pyrenoidosa* 820	Marine	Transcriptomic changes on different CO_2_ conditions	CO_2_	n.a	0.16% CO_2_ 0.04% CO_2_	Day 2 0.16% CO_2_ Day 2 0.04% CO_2_	[20]
*Chlorella *PY-ZU1	Freshwater	Carbon fixation mechanism of high CO_2_-tolerant *Chlorella*	Growth phase CO_2_	n.a	15% CO_2_ Ambient air	12 h, 24 h, 48 h, 72 h 15% CO_2_ 12 h, 24 h, 48 h, 72 h Ambient air	[13]
*Chlorella* sp. ABC-001	Marine	Growh specific changes in carbon fixation and lipid accumulation at high CO_2_ condition	Growth phase CO_2_	Total fatty acids by GC	10% CO_2_ Ambient air	Day 1, 3, 7, 10% CO_2_ Day 1, 3, 7, Ambient air	[This study]
*Chlorella zofingiensis* ATCC 30412	Freshwater	To identify genes and their expression level involved in astaxanthin and TAG biosynthesis	Carbon source	Total fatty acids by GC	Ambient air Glucose (glc)	6 h	[19]
*Chlorella vulgaris* UPSI-JRM01	Freshwater	Carbon partitioning toward starch and TAGs under nitrogen stress	Growth after nitrogen stress	Total fatty acids by GC	5% CO_2_	Day 1, 4, 8	[18]
*Chlorella* pyrenoidosa FACHB-10	Freshwater	To affirm the role of lipid turnover and identify differentially expressed transcription factors under nitrogen deprivation	Nitrogen	Fatty acids by GC–MS Lipid analysis by HPLC–MS	Ambient air	Day 2, 1.5 g/L nitrate Day 2, 0 g/L nitrate	[23]
*Chlorella* sp. C596	Marine	Transcriptomic changes before and after nutrient (nitrogen) deprivation	Nitrogen	TAGs by HPLC	15% CO_2_ for pH control	109 h, 192 h	[22]
*Chlorella sorokiniana* G32	Freshwater	To explain high starch accumulation and growth rate under mixotrophic condition	Glucose	Total lipid by gravimetric method	1.25 g/L glc 5 g/L glc	Before & After 1.25 g/L → 5 g/L glc Before & After 5 g/L → 1.25 g/L glc	[24]
*Chlorella* sp. TLD6B	Freshwater	To clarify the relationship between salt tolerance and lipid accumulation	Salt stress	Sulfo-phospho-vanillin fluorescence	Ambient air	18 h, 0 M NaCl 18 h, 0.1 M NaCl 18 h, 0.8 M NaCl	[25]
*Chlorella sorokiniana* UTEX 1602	Freshwater	DEGs in photosynthetic carbon fixation and lipid production under different nitrogen conditions and carbon source	Nitrogen Carbon source	Nile-red fluorescence	4% glc 4% CO_2_	48 h, 0.2% nitrate 48 h,0.8% nitrate 84 h, 0.2% nitrate, heterotrophic Day 8 0.033% nitrate, 4% CO_2_	[17]
*Chlorella* sp. BLD	Freshwater	Mechanisms of alkali resistance and lipid accumulation in the alkaliphilic microalgae *Chlorella* sp. BLD	pH	Total lipid by gravimetric method	Ambient air	Day 2 pH 10 Day 2 pH7.5	[36]
*Chlorella* sp. UMACC 237	Freshwater	Compare transcriptome profile of at ambient (4 °C) versus stress-inducing high (33 °C) temperatures	Temperature	n.a	Ambient air	120 h 4℃ 120 h 33℃	[26]
*Chlorella sorokiniana* NIES-2168	Freshwater	To elucidate the effects glucose assimilation and light intensity	Carbon source	n.a	Ambient air 2% glucose	Day 7 Autotrophic Day 7 Mixotrophic	[35]
*Chlorella vulgaris *PKVL 7422	Freshwater	To elucidate the effect of salt stress on metabolism and possible mechanisms of survival	Salt stress	n.a	Ambient air	Control wihout salt stress 2 h, 4 h NaCl 1% 2 h, 4 h NaCl 3%	[37]
*Chlorella* sp. L5	Freshwater	Revealing the phenol tolerance mechanism	Evolved strain for phenol	n.a	TAP	Day 4, L5 strain Day 4, L3 strain	[49]
*Chlorella pyrenoidosa* FACHB-5	Freshwater	Transcriptomic and Physiological Responses during Exposure to 17α-Ethinylestradiol	17α-Ethinylestradiol (17α EE_2_)	n.a	Ambient air	96 h 0 mg/L 17α EE_2_ 96,h 2 mg/L 17α EE_2_ 96 h 4 mg/L 17α EE_2_ 96 h 8 mg/L 17α EE_2_	[27]
*Chlorella *sp. Cv	Freshwater	To understand global response and possible tolerance mechanism against flue gas in an evolved strain	Flue gas	n.a	10% CO_2_	Day 1 flue gas Day 1 10% CO2	[28]

Photosynthesis with CO_2_ fixation is one of the most crucial bioprocesses in microalgae. Photosynthetic efficiency is highly affected by the concentration of the dissolved CO_2_ in a media. Huang et al*.* estimated the K_m_ value of RuBisCo (Ribulose-1,5-bisphosphate carboxylase) in *Chlorella* PY-ZU1 to be in the range of 80–192 μM, which exceeds the dissolved CO_2_ concentration (~ 15 μM) in water with air at an equilibrium state, indicating the necessity of CO_2_ concentrating mechanism (CCM) in microalgae [13, 31]. Carbonic anhydrase (CA) is the key enzyme for CCM, as it converts CO_2_ into more soluble HCO_3_^−^, allowing the dissipation of HCO_3_^−^ into the cell. Then, CA helps CO_2_ fixation by reconverting the internalized HCO_3_^−^ into CO_2_ at the thylakoid membrane under low CO_2_ concentrations. Hence, CA is highly expressed under CO_2_-limited conditions along with other CCM-related enzymes. Additionally, Jianhua et al. proposed several transporters related to CCM, which showed significant expression changes in the short term (0–24 h) by CO_2_ deprivation in *Chlorella pyrenoidosa* [21]. Besides, the previous transcriptomic profile of *C. pyrenoidosa* has also revealed 8 unigenes including ABC transporter, chlorophyll a/b binding protein, and photosystem I subunit V, which may play essential roles in CCM as well [20]. Although these findings have provided some information about CCM in *Chlorella* sp., there are still many unknown points underlying the CCM mechanisms.

The lipid accumulation mechanism in microalgae has been investigated over the years as lipids could be converted into biofuels or purified for human consumption (e.g. cooking oils, polyunsaturated fatty acids). In general, lipid production occurs primarily in the endoplasmic reticulum (ER) or chloroplast through the Kennedy pathway, storing energy in the form of triacylglycerol (TAG). Hence, many approaches for overexpression of Kennedy pathway genes including *glycerol-3-phosphate dehydrogenase (GPDH), glycerol-3-phosphate acyltransferase (GPAT), lysophosphatidic acid acyltransferase (LPAT), phosphatidic acid phosphohydrolase (PAP)*, and *diacylglycerol acyltransferase* (*DGAT*) has been reported, especially in oleaginous algae species such as *Phaeodactylum* and *Nannochloropsis* [32–34]. The lipid accumulation pathway in *Chlorella* sp. has also been extensively studied at the transcriptome level under various stress conditions [18, 35–37].

Most microalgal CO_2_ conversion studies have been conducted under moderately high CO_2_ conditions of 5% or below, and microalgae that grow well under 5–20% CO_2_ are considered CO_2_-tolerant strains [40]. *Chlorella* sp. ABC-001 showed enhanced growth under 10–15% CO_2_ conditions as compared to ambient air conditions [38, 39]. Thus, it is necessary to understand insights into the response of CO_2_-tolerant strain under high CO_2_ conditions for the use of industrial flue gases. Additionally, most studies focusing on lipid synthesis and transcriptomic analysis manipulate media composition or impose artificial stress to observe short-term changes. However, in large-scale cultivation, applying artificial stress is complex. This highlights the significance of studying natural nutrient depletion, an aspect that has received comparatively less attention in the field. Accordingly, it is meaningful to study the relationships between phenotypes and transcriptomic analysis according to CO_2_ and nutrient concentrations.

In this study, we conducted a time-course transcriptomic analysis of *Chlorella* sp. ABC-001, the high-CO_2_ tolerant strain, under ambient air and 10% CO_2_ conditions. We analyzed transcriptomic changes related to CO_2_ concentrations, cell growth phases, lipid accumulation, and nutrient depletion with a macroscopic perspective. Furthermore, we identified critical genes related to each metabolism and discussed them in detail. These findings are expected to enhance our understanding of the photosynthesis and lipid accumulation metabolism in the green alga *Chlorella* sp., especially under high CO_2_ conditions. Our study will also provide valuable insights into the metabolic engineering and synthetic biology of microalgae.

## Results

### Cultivation of ***Chlorella*** sp. ABC-001 under ambient air and 10% CO_2_ conditions

**Fig. 1:**
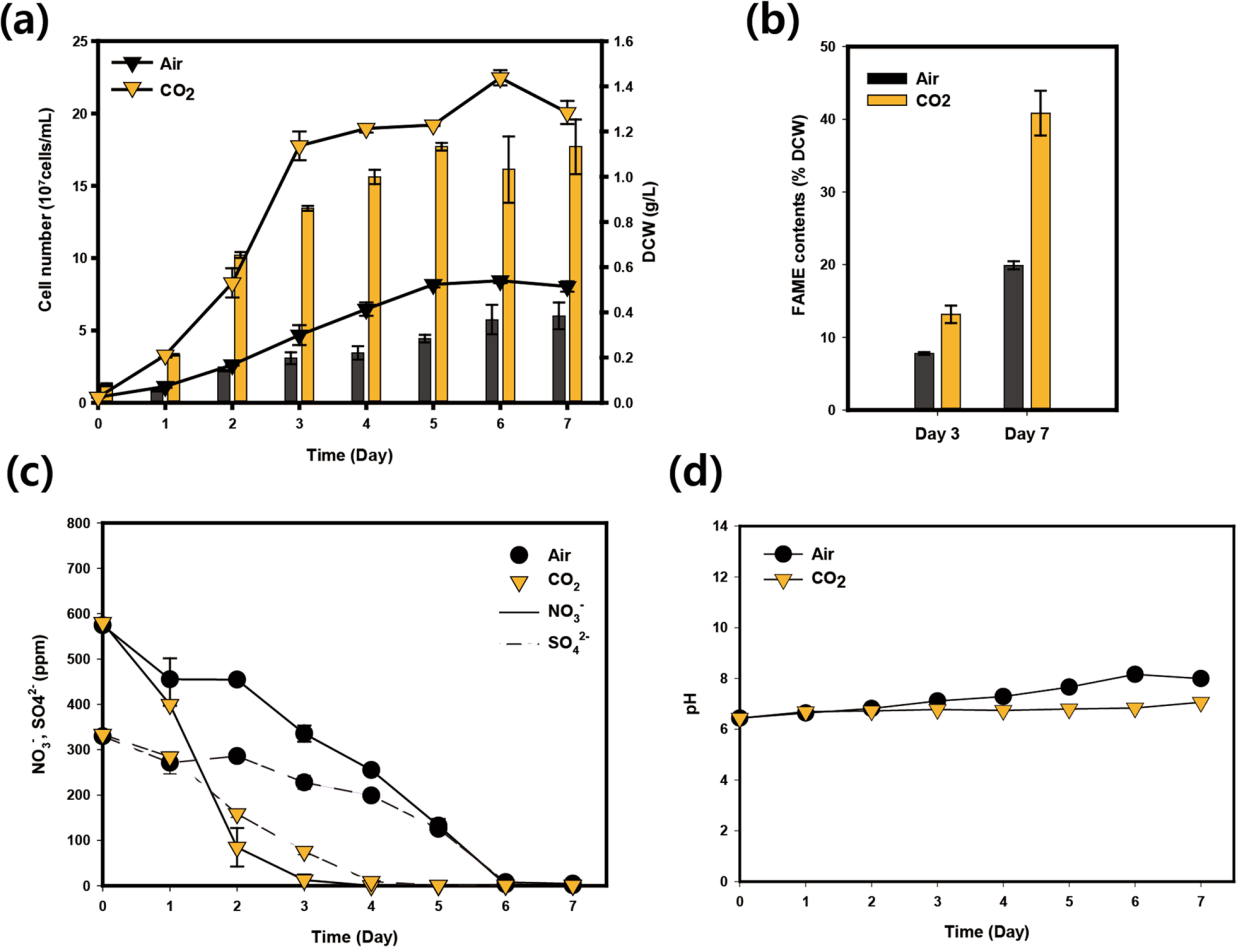
Cultivation of *Chlorella* sp. ABC-001 under different CO_2_ conditions. **a** Growth curve of cells cultivated with ambient air and 10% CO_2_. The line graph represents cellular density, and the bar graph represents dried cell weight. **b** The lipid contents of cells as measured after transesterification into FAME. **c** Changes in nitrate concentration (NO_3_^−^) and sulfate concentration (SO_4_^2−^). **d** pH changes. Error bars stand for the standard error calculated from three independent experimental data sets. Asterisks indicate the significant difference between cells cultivated under ambient air and 10% CO_2_ determined by Student’s *t*-test (*P < 0.05, **P < 0.01, ***P < 0.001)

In this study, triplicate biological cultivations of *Chlorella* sp. ABC-001 were carried out in different CO_2_ concentrations using photobioreactors for transcriptomic analysis. As described in Fig. 1, an apparent difference in growth and lipid accumulation was observed under the 10% CO_2_ condition compared to the ambient air condition. The maximum growth rate was faster with CO_2_ (μ_max_CO_2_: 0.84 d^−1^, μ_max_Air: 0.72 d^−1^), and the cells grew up to 2.5-fold higher cell density, accumulating almost twice more lipids (Fig. 1a, b). The nutrients were depleted faster in CO_2_ conditions, which was consistent with faster cell growth (Fig. 1c). Nitrogen and sulfur were almost depleted on day 3 and day 4 at 10% CO_2_, respectively, while it took 6 days to consume all these nutrients under ambient air conditions. The pH was maintained between 6 and 8 during the whole cultivation in both conditions, indicating that the effects of pH change may be negligible (Fig. 1d). Transcriptomic analysis through RNA sequencing was conducted to unravel the governing mechanisms and discover critical genes causing the phenotypic changes under different CO_2_ concentrations.

### RNA-sequencing and unigene assembly

The RNA samples were collected on days 1, 3, and 7 to represent the early-exponential phase, mid-exponential phase, and stationary phase under both ambient air and high CO_2_ conditions. For simplification, cells collected from each condition will be represented by ‘A1, A3, A7’ and ‘C1, C3, C7’ (A: ambient air, C: 10% CO_2_, 1: day1, 3: day3, 7: day7). The total number of 10–16 million (M) raw reads from the ambient air and high CO_2_ samples were sequenced, and 8–14 M clean reads were generated after trimming and sorting, as described in the material and method section. The clean read ratio of the raw sequence was about 87.75%, and the pairs plot analysis showed that the reproducibility of biological triplicate samples was higher than 0.9 in most cases, ensuring high reproducibility and reliability of the data (Additional file 1: Fig. S1). Clean reads were pooled for assembly, and a total of 10,246 annotated unigenes were generated, as summarized in Additional file 1: Table S1. The top 3 species represented in the National Center for Biotechnology Information (NCBI) non-redundant (NR) database were *Auxenochlorella protothecoides*, *Chlorella variabilis*, and *Coccomyxa subellipsoidea*, accounting for 52.7% of the transcripts (Additional file 1: Fig. S2). The whole transcriptomic data obtained in this study were deposited in the NCBI database under BioProject accession number PRJNA757763.

### Analysis of differentially expressed genes across different growth phases and CO_2_ concentrations

**Fig. 2:**
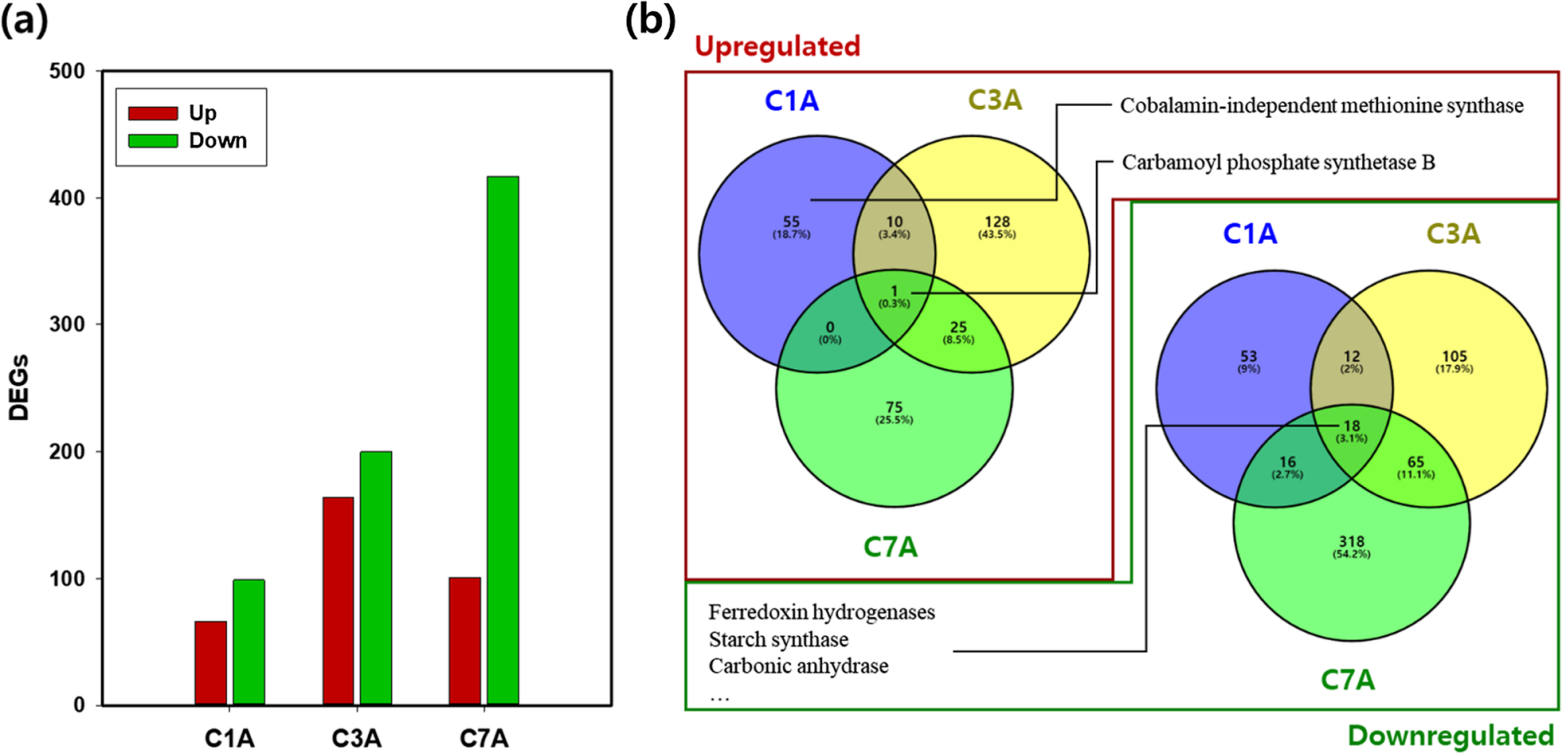
Transcript profiling changes by CO_2_ concentration. **a** The numbers of upregulated and downregulated DEGs in each condition. **b** Venn diagram representing the number of non-redundant DEGs from different CO_2_ concentrations. The left and right Venn diagram each represents upregulated and downregulated DEGs. C1A, C3A, and C3A represent the DEGs comparison of C1 vs A1, C3 vs A3 and C7 vs A7, respectively

Pairwise transcriptome comparison was performed to identify differentially expressed genes (DEGs). We compared expression levels under high CO_2_ conditions based on those under ambient air conditions on days 1, 3, and 7 (C1A: C1 vs A1, C3A: C3 vs A3, C7A: C7 vs A7). Basically, downregulated DEGs were more dominant than upregulated DEGs in all three comparisons under high CO_2_ conditions, and the number of downregulated DEGs also increased as the cultivation progressed (Fig. 2a).

Among all the identified DEGs, only one gene, carbamoyl phosphate synthetase B (Csorokiniana1SL000732t0001), showed a consistent upregulation in 10% CO_2_ during the whole cultivation period (Fig. 2b). Carbamoyl phosphate synthetase participates in the nitrogen metabolism pathway, catalyzing the first committed step in pyrimidine and arginine production [41]. Thus, we assume that the high expression level of carbamoyl phosphate synthetase was due to the faster nitrogen uptake in high CO_2_ conditions. The most notable upregulated gene was the cobalamin-independent methionine synthase (METE, Csorokiniana1SL000035t0006), which was observed in the C1A comparison. The expression level of METE was 20–32 fold higher in the C1 condition as compared to any other conditions including A1, A3, A7, C3, and C7 (Additional file 2). As METE is also involved in the synthesis of DNA, RNA, and protein, it seems to help with rapid growth in the early stage under high CO_2_ conditions.

There were 18 constitutively downregulated genes including carbonic anhydrase, ferredoxin hydrogenase, and starch synthase (Fig. 2b). The carbonic anhydrase and ferredoxin hydrogenase play essential roles in CCM [13]. Under the conditions of a sufficient external supply of carbon dioxide, the two genes might not need to be upregulated. Additionally, starch synthase was notably downregulated under high CO_2_ conditions as carbon storage is not preferred under favorable conditions. Indeed, the carbohydrate content of cells cultivated under ambient conditions was higher (40.7%) compared to those cultivated under 10% CO_2_ conditions (32.2%) on day 7 (Additional file 1: Fig. S3).

### Gene ontology analysis across different growth phases and CO_2_ concentrations

**Fig. 3:**
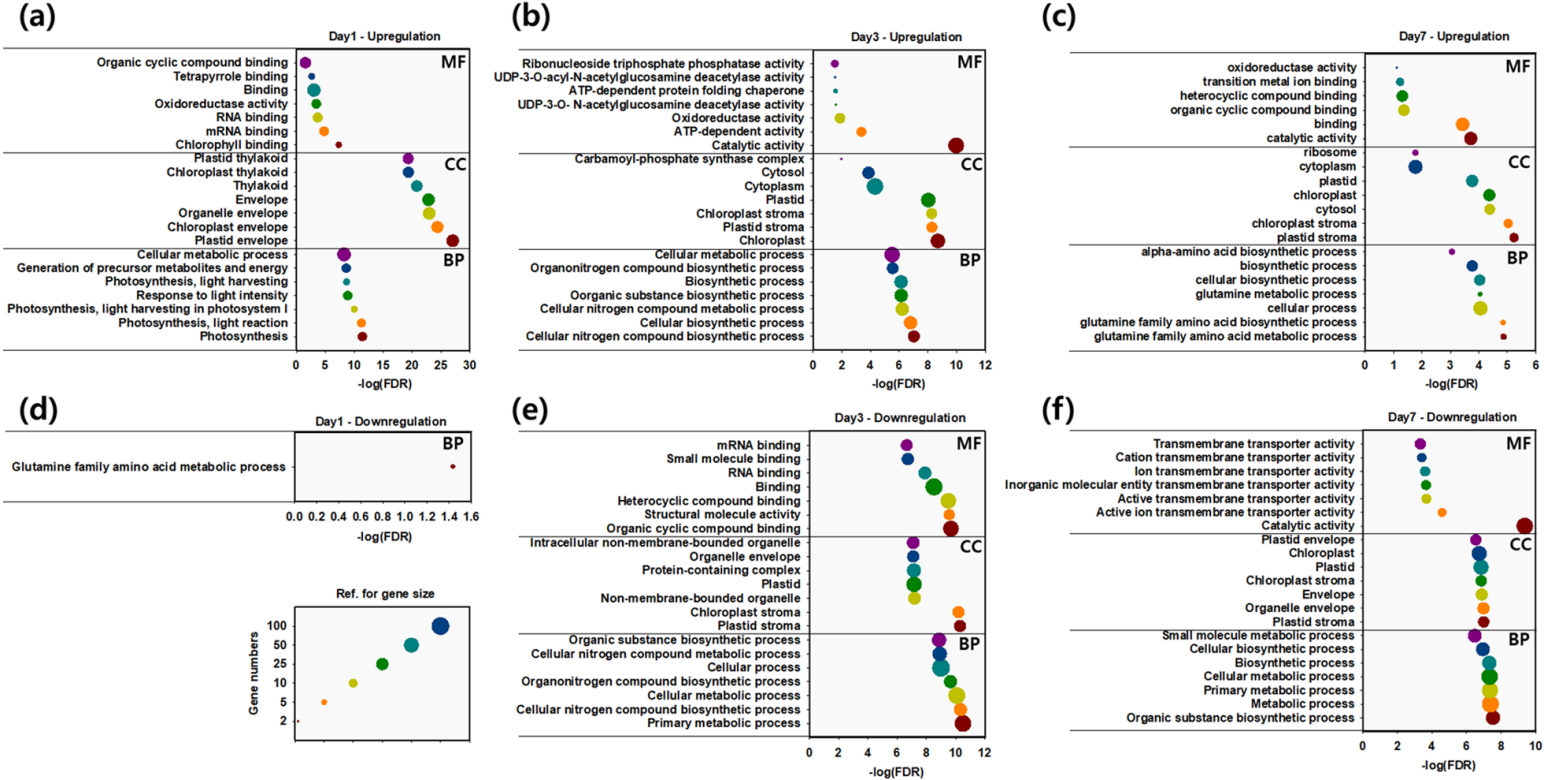
GO analysis of upregulated and downregulated non-redundant DEGs during cultivation under different CO_2_ concentrations. The top 7 terms were listed in each analysis based on the cellular growth phase of day 1 (**a**, **d**), day 3 (**b**, **e**), and day 7 (**c**, **f**). The size of the circle represents the gene numbers, and the reference is on the left bottom side of the figure

To further understand the function of the DEGs in each phase, Gene Ontology (GO) enrichment analysis was performed. Only the top 7 terms in each category of biological process (BP), cellular component (CC), and molecular function (MF) are listed (Fig. 3).

The early growth phase on day 1, the photosynthesis and energy-related genes in BP were mainly upregulated under high CO_2_ conditions (Fig. 3a). In CC, the genes of chloroplast-related organelles, such as the plastid, chloroplast, and thylakoid, were upregulated. In MF, the chlorophyll binding process is classified as the highly upregulated DEGs. On the other hand, there were no downregulated DEGs under high CO_2_ conditions on day 1, except for the glutamine metabolic process (Fig. 3d).

At the mid-exponential phase on day 3, more diverse changes were detected from both upregulated and downregulated DEGs (Fig. 3b and e). In MF, the cells primarily concentrated on protein-level regulation (including catalytic activity, oxidoreductase, chaperone, deacetylase, and phosphatase) under elevated CO_2_ conditions. Conversely, transcription-level regulation (such as RNA binding, small molecule binding, and mRNA binding) was downregulated. In addition, both upregulated and downregulated DEGs on day 3 were related to chloroplast and plastid stroma in the CC category. These findings suggest that the cells transitioned to the exponential growth phase on day 3. Indeed, *Chlorella* sp. ABC-001 exhibited accelerated growth under high CO_2_ conditions, and this might lead to a halt in the development of photosynthetic function while concentrating on a range of catalytic activities.

On day 7, a notable decrease in transmembrane transporter activity was observed in downregulated GO terms for MF under high CO_2_ conditions (Fig. 3f). This included diverse transporters such as metal transporters, ATP-binding cassette transporters, sugar transporters, and amino acid transporters (Additional file 2). As photosynthetic activity requires many membrane transporters [42], we expect the decreased photosynthetic activity may heavily cause the decreased membrane transporter activity. The ratio of OD_680_/OD_750_, which serves as an approximate indicator of photosynthetic efficiency and the physiological state of cells with their chlorophyll content [43], displayed a decreasing pattern under high CO_2_ conditions after day 3 (Additional file 1: Fig. S4). In contrast, cells grown under ambient air maintained a consistent OD_680_/OD_750_ ratio, further supporting our explanations.

### Transcriptomic profile analysis of CCM and the Calvin cycle

**Fig. 4:**
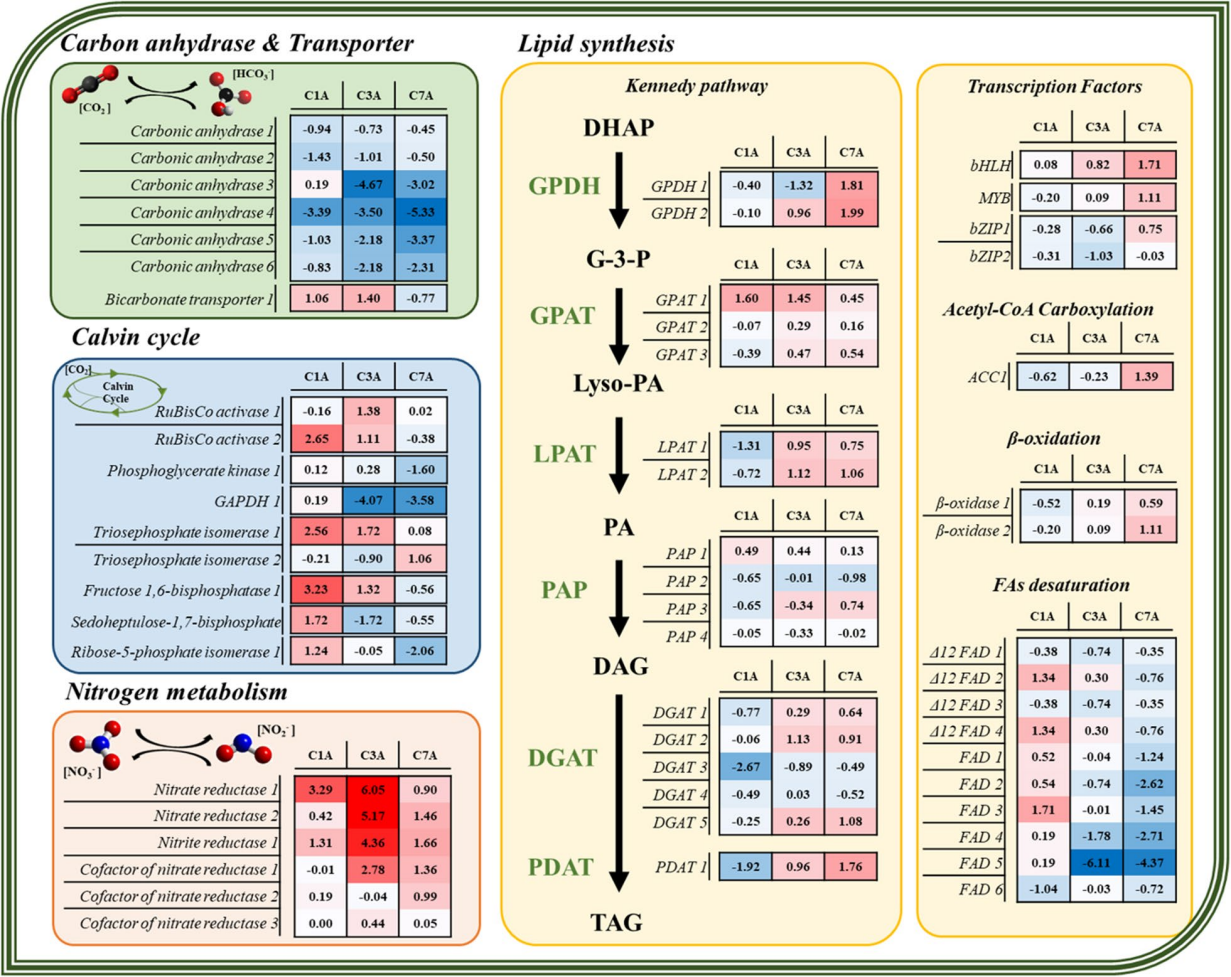
Relative expression levels (log_2_ fold change) of key enzymes participating in carbon fixation, Calvin cycle, nitrogen uptake, and lipid biosynthesis. The Gene IDs of each enzyme are listed in Additional file 1: Table S3. (*GAPDH* Glyceraldehyde-3-phosphate dehydrogenase, *GPDH* glycerol-3-phosphate dehydrogenase, *GPAT* glycerol-3-phosphate acyltransferase, *LPAT* lysophosphatidic acid acyltransferase, *PAP* phosphatidic acid phosphohydrolase, *DGAT* diacylglycerol acyltransferase, *PDAT* phospholipid-diacylglycerol acyltransferase, *bHLH* basic helix-loop-helix, *MYB* myeloblastosis, *bZIP* basic leucine zipper, *ACC1* Acetyl-CoA carboxylase 1, *FAD* Fatty acid desaturase)

To understand the mechanism behind CO_2_ fixation, growth, and lipid production under high CO_2_ conditions, we analyzed the associated transcriptomic profiles (Fig. 4). We first looked into the expression patterns of carbonic anhydrase (CA) genes and the Calvin cycle-related genes (Fig. 4). In *Chlorella* sp. ABC-001, 6 CAs were identified, and the CAs generally showed lower expression levels under CO_2_-supplemented conditions. Particularly, the CA3 and CA4 showed the most significant changes, 25–40-fold lower expression levels under high CO_2_ conditions than under ambient air conditions. This means that the CAs, especially CA3 and CA4, are not necessary under high CO_2_ conditions. Conversely, they are expected to play a critical role in the carbon concentrating mechanism under low CO_2_ conditions in *Chlorella* sp. ABC-001. The bicarbonate transporter 1, which is an essential transporter for carbon fixation, showed higher expression in high CO_2_ conditions on days 1 and 3.

The expression patterns of two potential RuBisCo activases were also analyzed. The RuBisCo activase 1 and 2 showed higher expression levels on day 3 under high CO_2_ conditions. In particular, the RuBisCo activase 2 was significantly upregulated on day 1 at the early growth phase. On day 1, most of the genes in the Calvin cycle showed higher expression levels in the CO_2_-supplemented culture, whereas it became the opposite on day 7.

### Transcriptomic profile analysis of lipid synthesis

In microalgae, nitrogen limitation is the most effective stress to trigger lipid accumulation in the cell [44]. According to Fig. 1c, nitrogen starvation was initiated after day 3 and day 6, under 10% CO_2_ and ambient air conditions, respectively. From the RNA-seq comparison of CO_2_ vs ambient air, a sudden increase of expression level in nitrate reductase (NR), nitrite reductase (NiR), and cofactor of nitrate reductase (CoNiR) were found on day 3, when nitrogen was almost depleted in 10% CO_2_ conditions (Fig. 4). The expression levels of NR, NiR, and CoNiR also consistently increased from day 1 to day 7 in both cultivation conditions (Additional file 1: Fig. S5). High expression levels of NR after nitrogen depletion at the mid-exponential phase have been reported by other researchers as well [13].

Microalgae generally accumulate lipids in the form of tri-acyl-glycerol (TAG), which is synthesized via the Kennedy pathway. Under high CO_2_ conditions, FAME contents were almost 40% on day 7, which was 3.1-fold higher than those on day 3 (Fig. 1b). The gene expression patterns of the Kennedy pathway were also related to the phenotypes. The genes encoding for glycerol-3-phosphate dehydrogenase (GPDH), lysophosphatidic acid acyltransferase (LPAT), diacylglycerol acyltransferase (DGAT), and phospholipid-diacylglycerol acyltransferase (PDAT) showed an increased expression level under high CO_2_ conditions on day 3 and 7. However, PAP seemed not to be affected much by CO_2_ concentrations. A distinct decrease in the expression level of *GPDH*, *LPAT*, *PAP*, *DGAT*, and *PDAT* was observed under 10% CO_2_ conditions on day 1. As C1 is the condition that exhibited a very active metabolism in photosynthesis and energy generation for growth compared to A1 (Figs. 1a and 3a), lipid synthesis seems to be strictly repressed at this point.

In particular, a putative *PDAT* (Csorokiniana1SL007740t0001) and *GPDH* genes showed the most noticeable changes, which increases the likelihood of its correlation with lipid accumulation. PDAT, similar to DGAT, delivers an acyl group to diacylglycerol (DAG) in the last step of TAG biosynthesis. However, unlike DGAT, which takes acyl-group from acyl-CoA, PDAT functions on diverse kinds of substrates such as phospholipids (phosphatidylcholine, phosphatidylenolamine), membrane lipids (monogalactosyl diacylglycerol, digalactosyl diacylglycerol), and DAG [45]. Accordingly, GPDH and LPAT are also expected to be important for lipid accumulation according to their increased expression pattern.

We have broadened our analysis to explore additional factors and pathways involved in lipid synthesis in microalgae, not limited to the Kennedy pathway (Fig. 4). Among the transcription factors (TFs) known to promote lipid synthesis in microalgae, basic helix-loop-helix (bHLH) [46] and myeloblastosis (MYB) [47] TFs displayed increased expression profiles under high CO_2_ conditions, which correlated with lipid accumulation. In contrast, basic leucine zipper (bZIP) TFs [48] did not exhibit a clear relationship with lipids. Acetyl-CoA carboxylase and β-oxidizing enzymes, responsible for the synthesis and cleavage of acyl-CoA, respectively, showed elevated expression levels under 10% CO_2_ conditions during the stationary phase. Additionally, we identified multiple potential fatty acid desaturase (FAD) genes; all 10 FADs consistently demonstrated lower expression levels under high CO_2_ conditions on day 7.

### Validation of transcriptomic analysis via qRT-PCR

**Fig. 5:**
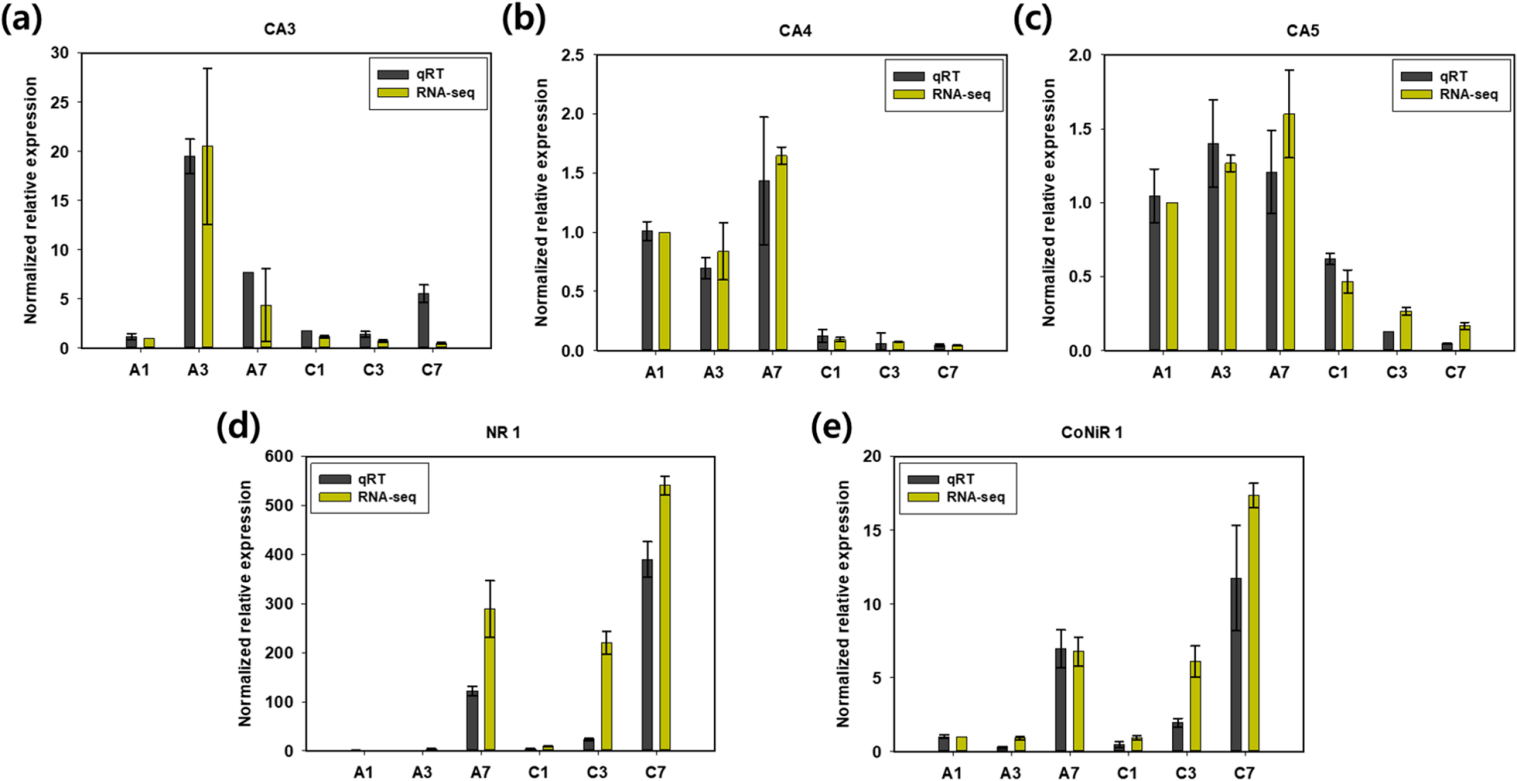
Validation of RNA-sequencing data by qRT-PCR. Each data was normalized by the expression level of A1. Error bars stand for the standard error calculated from three independent experimental data sets. Specific gene IDs are listed in Additional file 1: Table S3

To validate the quality of the RNA-sequencing data obtained in this study, qRT-PCR was carried out by targeting several genes exhibiting different expression patterns (Fig. 5). Indeed, the qRT-PCR results were almost consistent with the RNA-sequencing data. CA3, CA4, and CA5 showed low expression levels under high CO_2_ conditions and high expression levels under ambient air conditions by qRT-PCR. Although the expression level of NR1 was lower in qRT-PCR compared to RNA-seq, the overall expression pattern was consistent in both analyses. The CoNiR1 also showed highly conserved expression patterns between qRT-PCR and RNA-sequencing results. Based on these results, we confirmed the credibility of the RNA-sequencing analysis carried out in this study.

## Discussion

As the potential of *Chlorella* for CO_2_ fixation and production of lipids/high value-added substances has been highlighted, a number of transcriptomic approaches have been conducted to elucidate the underlying mechanisms in recent days [13, 17–21, 49, 50]. These researches covered a broad range of areas such as hydrogen photoproduction, TAG synthesis, stress tolerance, and starch synthesis. Due to the dynamic activity of cellular metabolism and the formidable amount of transcriptomic data, various interpretations are possible depending on sampling points, comparison groups, and analytic criteria.

**Fig. 6:**
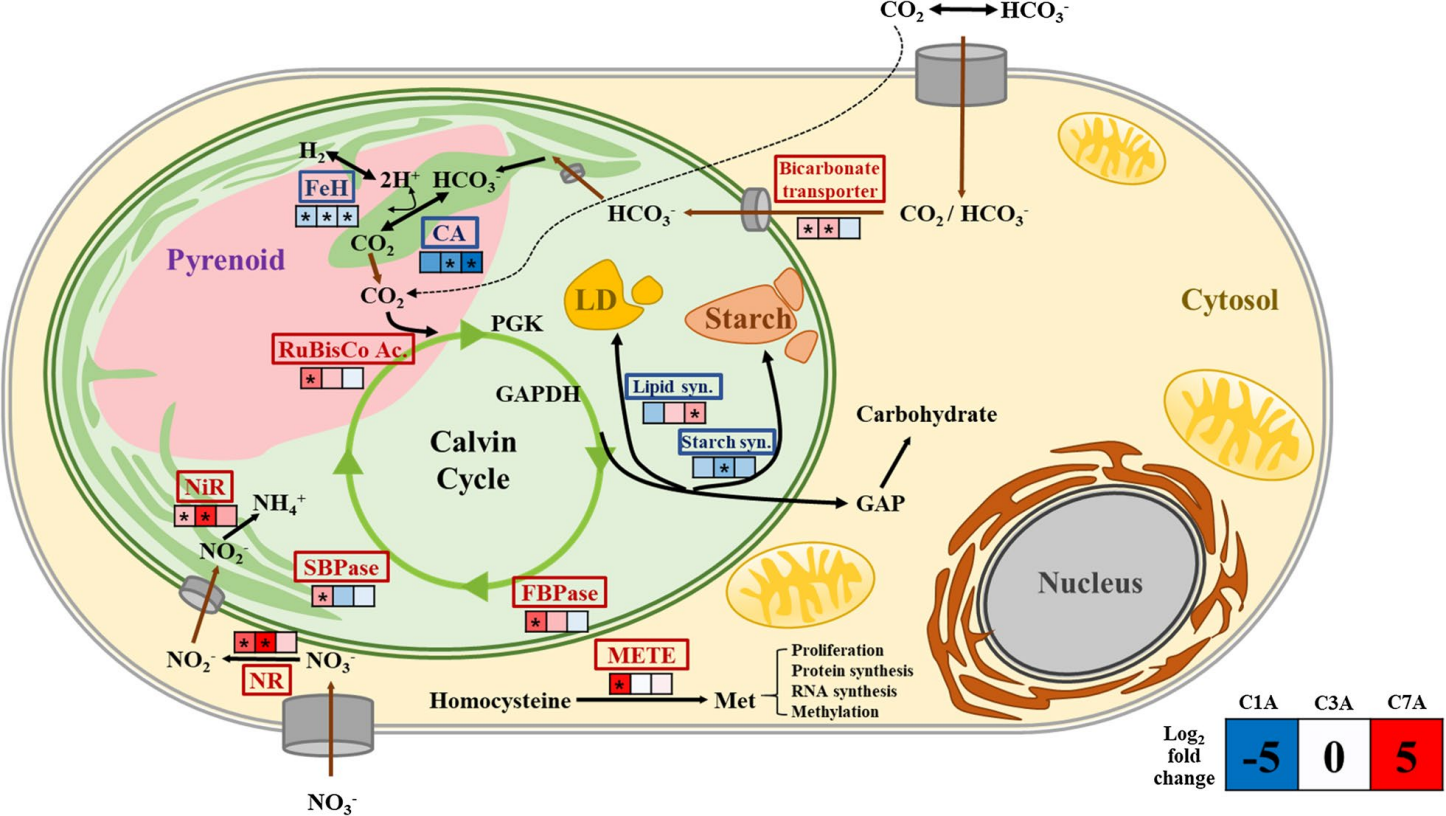
Overall metabolic changes in *Chlorella* sp. ABC-001 at different CO_2_ environments. The color of each enzyme represents either upregulation (red) or downregulation (blue) of genes on day 1. Small boxes stand for the log2 fold changes of representative enzymes of each metabolic reaction. The asterisk indicates adj.*p* < 0.05. Specific gene IDs are listed in Additional file 1: Table S3. (*FeH* ferredoxin hydrogenase, *SBPase* sedoheptulose-1,7-bisphosphate, *FBPase* fructose 1,6-bisphosphatase, *GAPDH* glyceraldehyde-3-phosphate dehydrogenase, *CA* carbonic anhydrase 4, *NR* nitrate reductase1, *NiR* nitrite reductase1, *METE* cobalamin independent methionine synthase, *Met* methionine, *Lipd syn* represented by PDAT1, *Starch syn* represented by SS)

In this study, we performed a transcriptomic analysis of *Chlorella* sp. ABC-001 under different CO_2_ concentrations (10% CO_2_ and ambient air) at three different time points (early exponential, mid-late exponential, and stationary growth phase). From transcriptomic analysis, a total of 10,246 putative genes were annotated, which is an improved result compared to previous studies (9590) on transcriptome profiling of *C. pyrenoidosa* in response to different CO_2_ concentrations [20]. Based on the transcriptome data, we illustrated the central carbon metabolism of *Chlorella* sp. ABC-001 with their expression levels at high CO_2_ conditions as compared to ambient air conditions according to the time point in Fig. 6.

During the early growth stage under high CO_2_ and nutrient conditions, we observed that a high CO_2_ level boosted the construction of photosynthetic organelles at the early stage and upregulated the genes related to the Calvin cycle and bicarbonate transporter to incorporate the inorganic carbon (Figs. 3, 4, and 6). At the same time, nitrate/nitrite reductases were also upregulated as nitrogen is an essential component for chlorophylls under high CO_2_ conditions. At the early growth stage (day 1), cells prioritize proliferation while inactivating energy-storage processes, including lipid/starch synthesis and the carbon concentrating mechanism. The intensive expression level of a cobalamin-independent methionine synthase (METE) at the early stage under high CO_2_ conditions also supports this idea. METE is the only enzyme that can synthesize methionine in an environment without additional vitamin B_12_ (cobalamin) [51]. The synthesized methionine is used for protein synthesis and also plays an important role in proliferation by methylation of DNA and RNAs [52, 53]. Fast nitrogen uptake may also be related to methionine synthesis because nitrogen is known to enhance the METE activity by 30–40 folds (Fig. 1c) [54].

From the middle of the exponential phase, cells started downregulating photosynthetic activity under high CO_2_ conditions. Particularly, the cells deactivated CCM and decreased the expression level of carbonic anhydrases because they do not need to use tremendous energy for CO_2_ transport under high CO_2_ conditions. We also found the low expression level of ferredoxin hydrogenase 4 (FeH) located in the chloroplast under high CO_2_ conditions (Additional file 2). The FeH might catalyze the production of hydrogen ions which is necessary for the reduction of bicarbonate and CO_2_ supplement. We also found the upregulation of NR, NiR, and CoNiR according to nitrogen limitation under 10% CO_2_ conditions, indicating that the cells subsequently would focus on lipid synthesis.

*Chlorella* sp. ABC-001 changed gene expression patterns for energy storage in lipids along with nutrient depletion and CO_2_ concentrations at the mid-exponential and stationary phases. Indeed, the variations in lipid composition observed in our study cannot be solely attributed to the direct influence of CO_2_ concentration. Instead, these results likely represent a complex array of interacting factors. The early onset of nitrogen starvation was primarily instigated by the high CO_2_ concentration, contributing to the change in the expression of lipid-related genes. Furthermore, shading effects resulting from high cell concentrations may also play a part in interacting with photosynthetic activity. According to the gene expression patterns under high CO_2_ conditions, GPDH and PDAT seemed to play important roles in lipid synthesis. *GPDH1*, *GPDH2*, and *PDAT* were downregulated at the early growth phase and highly upregulated at the stationary phase, where lipid was significantly accumulated (Fig. 4). Indeed, Yao et al*.* reported that the overexpression of endogenous GPDH in *Phaeodactylum tricornutum* resulted in a 60% increase in neutral lipids [55]. *PDAT* usually exists as a single-copy gene, even in oleaginous strains [56] and it has been suggested that PDAT contributes to TAG synthesis in *Chlamydomonas reinhardtii*, *Myrmecia incisa and Nannochloropsis* [45, 57, 58]. Recently, engineering of PDAT was actually proven to be effective for increasing the TAG contents in *Nannochloropsis* [58, 59]. The fatty acid composition of lipids also supported the transcriptomic and phenotypic data (Additional file 1: Fig. S6). On day 7, α-linolenic acid (C18:3) decreased, while oleic acid (C18:1) increased under 10% CO_2_ conditions. The change in fatty acid composition might be primarily attributed to the consistently lower expression levels of multiple FADs (Fig. 4). Additionally, the degradation of chloroplasts may contribute to the fatty acid composition, as polyunsaturated fatty acids are the primary components of membrane lipids [60, 61]. Consequently, fatty acids and acyl-groups from degraded membrane lipids, which correspond well with increased β-oxidase expression, can be used for de novo TAG synthesis by PDAT. Thus, the putative *PDAT* gene (Csorokiniana1SL007740t0001) showing a high expression level at the lipid accumulation stage in *Chlorella* sp. ABC-001 can be a promising genetic target for lipid production in microalgae.

Generally, *DGAT* genes have been studied well in microalgae because DGAT is in charge of the last step for lipid synthesis, transferring the acyl group to DAG for TAG synthesis. *DGAT* genes often exist with multiple copies in microalgae, and only some of them were proven to be effective in enhancing lipid accumulation [62, 63]. We also identified 5 *DGAT* genes in *Chlorella* sp. ABC-001. However, their expression patterns were not consistent with the lipid accumulation phenotype. Although the expression patterns of *DGAT1*, *DGAT2*, and *DGAT5* are similar to the lipid accumulation pattern, the expression levels were not as high as that of *GDPH* and *PDAT*. This pattern was similar to *GPAT* and *LPAT*. Thus, The *DGAT*, *GPAT*, and *LPAT* from *Chlorella* sp. ABC-001 might not be suitable target genes for lipid synthesis.

Taken together, our analysis successfully elucidated the relationship between the phenotypic and transcriptomic changes of *Chlorella* sp. ABC-001 in response to CO_2_ conditions across various growth phases. Moreover, we identified key genes involved in carbon concentration mechanism (CCM), cell development, and lipid accumulation under high CO_2_ conditions. This study provides high-quality transcriptomic data that can be applied in microalgal synthetic biology and metabolic engineering for industrial purposes, particularly in utilizing industrial exhaust gas containing high CO_2_ concentrations. Our time-course transcriptomic analysis (days 1, 3, and 7) addresses a gap in the current research landscape, shedding light on the molecular mechanisms underlying long-term adaptations. While many studies focus on shorter time frames (24 or 48 h), we deliver a more comprehensive understanding of metabolic changes and adaptations over extended periods, better representing industrial microalgal cultivation conditions.

## Conclusions

To address climate change issues, it is crucial to research the production of high-value materials utilizing industrial flue gases. However, conventional microalgae typically do not have high CO_2_ tolerance. In this study, we analyzed the transcriptome of *Chlorella* ABC-001, a high CO_2_ tolerant strain, over an extended period (7 days) during which considerable lipid accumulation occurred under high CO_2_ conditions. We confirmed that *Chlorella* sp. ABC-001 focuses on fast growth at the early stage under high CO_2_ conditions by upregulating the Calvin cycle, METE, and bicarbonate transporters. However, CA expression was decreased because CCM did not need to be significantly activated in the high CO_2_ supply. In addition, our transcriptomic analysis revealed that PDAT and GPDH, the first and final genes for TAG synthesis, are expected to play a crucial role in lipid synthesis under high CO_2_ conditions. This in-depth knowledge can contribute to developing more efficient microalgal cultivation strategies and innovative genetic engineering approaches for enhancing growth and lipid production capabilities.

## Materials and methods

### Strains and cultivation

*Chlorella* sp. ABC-001, a marine microalgae isolated from a coastal region in Jeju island (South Korea) was used in this study [39]. This species was chosen for its high CO_2_ tolerance, and the transcriptomic differences were analyzed under both aerobic and 10% CO_2_-supplied conditions [38]. The cells were maintained and cultivated in N8 media (1 g L^−1^ KNO_3_, 0.74 g L^−1^ Na_2_HPO_4_·2H_2_O, 13 mg L^−1^ CaCl_2_·2H_2_O, 10 mg L^−1^ FeEDTA, 50 mg L^−1^ MgSO_4_·7H_2_O, 12.98 mg L^−1^ MnCl_2_·4H_2_O, 3.2 mg L^−1^ ZnSO_4_·7H_2_O, 1.83 mg L^−1^ CuSO_4_·5H_2_O, 3.58 mg L^−1^ Al_2_(SO_4_)_3_·18H_2_O) through the whole experiments. The cultivation was performed in 500 mL column PBRs (photobioreactors) with working volumes of 400 mL under 200 μmol photons m^−2^ s^−1^ of light at 30 ℃. The initial concentrations in each experiment were fixed at 5 $\times$ 10^6^ cells mL^−1^. For aerobic conditions, ambient air was supplied at 0.6 vvm (volume gas per volume medium per minute), whereas 0.6 vvm of 10% CO_2_ was supplied for CO_2_-sufficient conditions.

### Cultivational analysis

The growth of cells was measured daily in terms of cell density and dried cell weight (DCW) using a Cellometer (Auto X4, Nexcelom, USA) and filter paper (Whatman, USA) as described in previous publications [64]. The optical densities at wavelengths of 680 nm and 750 nm were measured using a UV–VIS spectrophotometer (UV-1800, Shimadzu, Japan). Accumulated lipids were determined by gas-chromatography (HP 6890; Agilent Technologies, CA, USA) after extraction and transesterification through the Bligh-Dyer method similar to our previous research [65]. The remaining nutrients in the media, particularly nitrate and sulfate, were measured with an elemental analyzer (FLASH 2000 series; Thermo Scientific, MA, USA) at the KAIST Analysis Center for Research Advancement [66]. The total carbohydrate content was quantified using the phenol–sulfuric acid method described in a previous study [38]. In brief, approximately 3–5 mg of dried biomass powder was weighed, thoroughly mixed in 10 mL of distilled water, and then 1 mL of the mixture was transferred to clean glass tubes. Next, 1 mL of 5% wt. phenol solution and 5 mL of concentrated sulfuric acid were added, and the tubes were left in the dark for 30 min to facilitate the reaction. The tubes were inverted several times to mix the solution well, and the absorbance at 470 nm was measured using a UV/Vis spectrophotometer (Shimadzu, Japan).

### Transcriptomic analysis

#### RNA extraction, RNA sequencing, and annotations

The cultivated cells were harvested on days 1, 3, and 7 in each condition and were homogenized with a bead beater (Percellys 24, Bertin Technologies, Paris, France). Using NucleoZOL (Macherey–Nagel, Düren, Germany) according to the provided protocol, RNA was extracted from the homogenized cells. gDNA was then removed from the collected RNAs using DNA-freeTM DNase kits (Ambion, TX, USA). Finally, cDNA was synthesized using an oligo (dT) 20 primer (Invitrogen, CA, USA) and Superscript III^™^ Reverse Transcriptase (Invitrogen, CA, USA).

RNA-seq was conducted using an Illumina HiSeq2500, and 18 FASTQ files of sequences were yielded (A1, A3, A7, C1, C3, C7, three biological replicates per condition). Then, the transcriptome short reads were pre-treated using DynamicTrim and LengthSort provided by the Solexa QA package to sort out low-quality reads. The resulting clean-reads were assembled by Velvet (ver. 1.2.08) and Oases (ver.0.2.08) according to the provided protocols [67, 68]. The quality of RNA was assured by statistics of dynamic trim (Additional file 3), length sort (Additional file 3), final assembled transcripts (Additional file 3), and RNA integrity electropherograms (Additional file 1: Fig. S7). Annotation was conducted using BLASTX against amino acids from a diverse database including Phytozome v9, NCBI NR, Uniprotkb, KOG, and KEGG (*e* ≤ 1e^−10^). InterProscan was carried out using tools provided by EMBL as the default option.

#### Analysis of DEGs

For the selected clean reads, the read counts were calculated through mapping using Bowtie2 (v2.1.0) software (mismatch < 2 bp, penalty) [69]. Samples with high deviations were normalized by DEseq using R (Additional file 1: Fig. S8) [70]. Differentially expressed genes (DEGs) of *Chlorella* sp. ABC-001 during cultivation were selected according to the twofold change method and binomial method, where the criteria were set as ‘adjusted *P*-value (FDR) < 0.01’. When the log_2_ value of the fold change was higher than 1, it was annotated as an ‘up-regulation’, and when the value was lower than -1, it was annotated as a ‘down-regulation’. For GO (gene ontology) analysis, the selected DEGs were aligned to the sequences from the GO database and processed through Blast2GO. The significance level was set at 0.05 and categorized into three functional categories of BP (biological process), CC (Cellular component), and MF (molecular function). DEGs were annotated by BLASTX (*e* ≤ 1e^−10^, Best hits) against amino acid sequences from KEGG DB. The selected DEGs are described by the MA plot in Additional file 1: Fig. S9.

#### Quantitative real-time PCR (qRT-PCR) validation

The cDNA prepared for RNA sequencing was also used as a qRT-PCR template for validation. The housekeeping gene for TATA-box binding protein (*TATA*) was used as a control. All the primers used for qRT-PCR are listed in Additional file 1: Table S2. qRT-PCR was performed with 20 μL reaction volumes containing 2 μL of cDNAs (20 ng), 0.5 μL of each primer (10 μM), and 10 μL of Universal SYBR supermix (Bio-Rad, USA). The mixture was loaded on 96-well Hard Shell PCR plates (Bio-Rad, USA) and analyzed using a CFX96 Real-Time system (Bio-Rad, USA).

### Statistic analysis

Three biological replicates were performed for all experiments, with results presented as mean ± standard error (SE). Statistical analysis was performed using an in-house script for DEG selection and metaboanalyst 5.0 (*t*-test, FDR) for other experimental data [71]. Detailed explanations for each case are described in the “Analysis of DEGs” section and legends of each figure.

## Supplementary Information

**Additional file 1: Fig. S1–S9**, **Tables S1–S3**. **Fig. S1** Pairs plot analysis for examining the reproducibility of data sets. **a** A1D, **b** A3D, **c** A7D, **d** C1D, **e** C3D, **f** C7D. **Fig. S2** Gene ratio by species of genes used to annotate representative transcripts using NCBI NR. **Fig. S3 **Carbohydrate content of cells cultivated under ambient air conditions and 10% CO_2_ conditions on day 7. **Fig. S4 **The changes in OD680/OD750 during the whole cultivation period. The ratio of OD680/OD750 is an approximate indicator of the photosynthetic efficiency and the physiological state of the cells in terms of their chlorophyll contents. **Fig. S5** Relative expression levels (log_2_ fold change) of key enzymes participating in carbon fixation, Calvin cycle, nitrogen uptake, and lipid biosynthesis. Time-course comparison of cells cultivated in the same CO_2_ concentration (10% CO_2_ or ambient air) at different growth phase. **Fig. S6** The profiles of fatty acid composition of cells under A3D, A7D, C3D, and C7D conditions. Error bars stand for the standard error calculated from three independent experimental data sets. **Fig. S7** The RNA integrity electropherograms of each sample. **Fig. S8** Boxplot of gene expression level before and after normalization. **a** Average expression level of the raw data from each sample **b** Average expression level of the normalized data from each sample. **Fig. S9** MA plot of DEGs in each condition. X-axis represents the average expression value of the control and sample on a log_2_ scale, and the Y-axis represents the fold change between the two samples, on a log_2_ scale.** Table S1** Statistical results of annotated final unigenes. **Table S2** Primers used in this study for qRT-PCR. **Table S3** Gene IDs of key enzymes described in Figs. 4, 5 and 6.

**Additional file 2: **Transcription levels of unigenes and DEGs.

**Additional file 3: **Detailed information of transcriptome sequencing data.

## Data Availability

The datasets generated during the current study are available at NCBI under BioProject accession code PRJNA757763.

## Abbreviations

GO: Gene ontology
BP: Biological process
CC: Cellular component
MF: Molecular function
DEG: Differentially expressed genes
CCM: Carbon concentrating mechanism
FDR: False discovery rate
CA: Carbonic achydrase
GPDH: Glycerol-3-phosphate dehydrogenase
GPAT: Glycerol-3-phosphate acyltransferase
LPAT: Lysophosphatidic acid acyltransferase
PAP: Phosphatidic acid phosphohydrolase
DGAT: Diacylglycerol acyltransferase
PDAT: Phospholipid-diacylglycerol acyltransferase
GAPDH: Glyceraldehyde-3-phosphate dehydrogenase
RuBisCo: Ribulose-1,5-bisphosphate carboxylase
TAG: Triacylglycerol
DAG: Diacylglycerol
METE: Cobalamine-independent methionine synthase
bHLH: Basic helix-loop-helix
MYB: Myeloblastosis
bZIP: Basic leucine zipper
ACC1: Acetyl-CoA carboxylase 1
FAD: Fatty acid desaturase
