# Supplementary material for: Transcriptional insights into Chlorella sp. ABC-001: a comparative study of carbon fixation and lipid synthesis under different CO2 conditions
Source: Biotechnol Biofuels Bioprod. 2023 Jul 15;16:113. doi: 10.1186/s13068-023-02358-4 (PMC10350272; doi:10.1186/s13068-023-02358-4)
Supplement: Supplementary file 1 — Additional file 1: Fig. S1–S9, Tables S1–S3. Fig. S1 Pairs plot analysis for examining the reproducibility of data sets. a A1D, b A3D, c A7D, d C1D, e C3D, f C7D. Fig. S2 Gene ratio by species of genes used to annotate representative transcripts using NCBI NR. Fig. S3 Carbohydrate content of cells cultivated under ambient air conditions and 10% CO2 conditions on day 7. Fig. S4 The changes in OD680/OD750 during the whole cultivation period. The ratio of OD680/OD750 is an approximate indicator of the photosynthetic efficiency and the physiological state of the cells in terms of their chlorophyll contents. Fig. S5 Relative expression levels (log2 fold change) of key enzymes participating in carbon fixation, Calvin cycle, nitrogen uptake, and lipid biosynthesis. Time-course comparison of cells cultivated in the same CO2 concentration (10% CO2 or ambient air) at different growth phase. Fig. S6 The profiles of fatty acid composition of cells under A3D, A7D, C3D, and C7D conditions. Error bars stand for the standard error calculated from three independent experimental data sets. Fig. S7 The RNA integrity electropherograms of each sample. Fig. S8 Boxplot of gene expression level before and after normalization. a Average expression level of the raw data from each sample b Average expression level of the normalized data from each sample. Fig. S9 MA plot of DEGs in each condition. X-axis represents the average expression value of the control and sample on a log2 scale, and the Y-axis represents the fold change between the two samples, on a log2 scale. Table S1 Statistical results of annotated final unigenes. Table S2 Primers used in this study for qRT-PCR. Table S3 Gene IDs of key enzymes described in Figs. 4, 5 and 6. [file 13068_2023_2358_MOESM1_ESM.docx]

Additional File 1

Additional file Figures


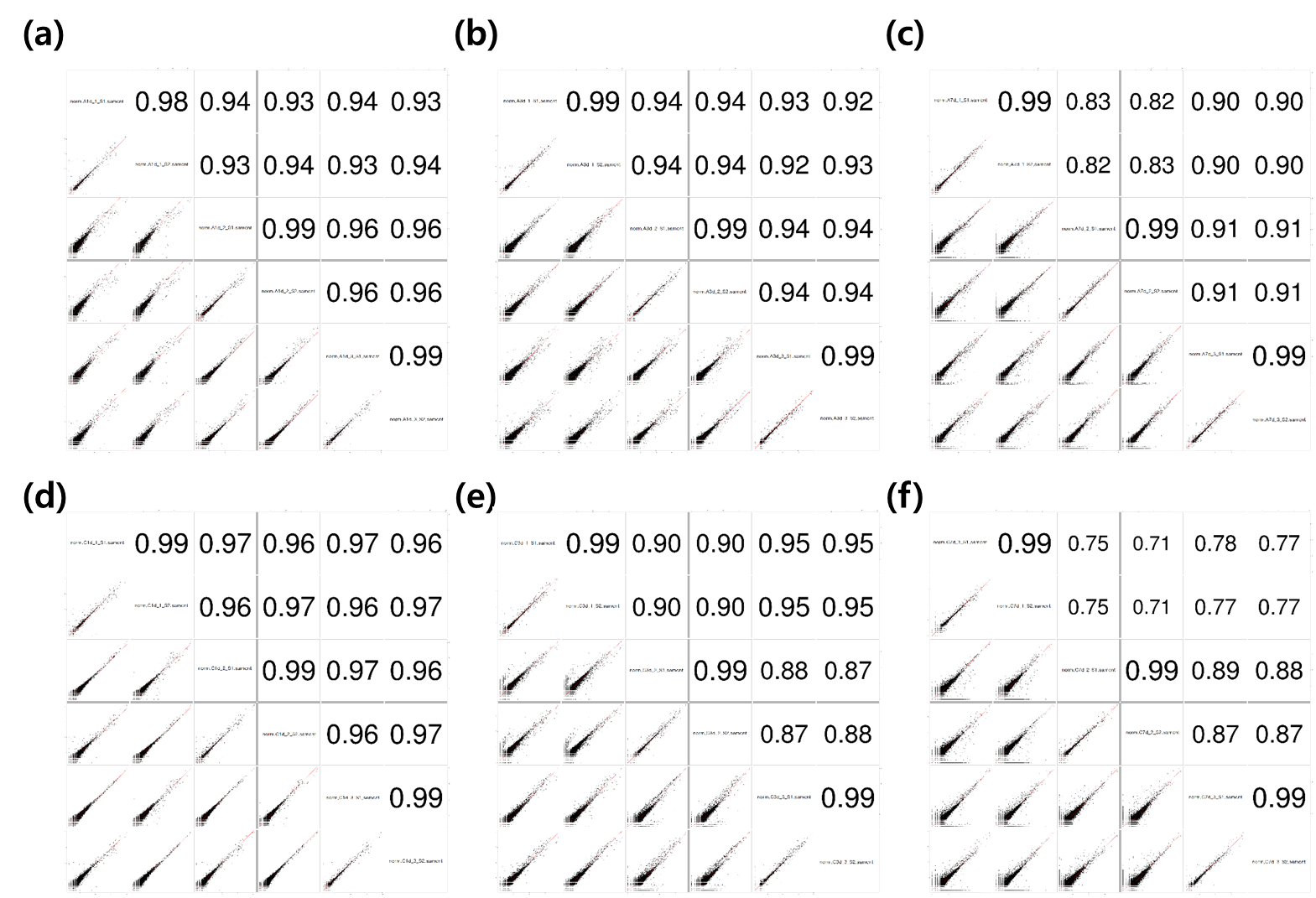


**Fig S1.** Pairs plot analysis for examining the reproducibility of data sets. (a) A1D, (b) A3D, (c) A7D, (d) C1D, (e) C3D, (f) C7D


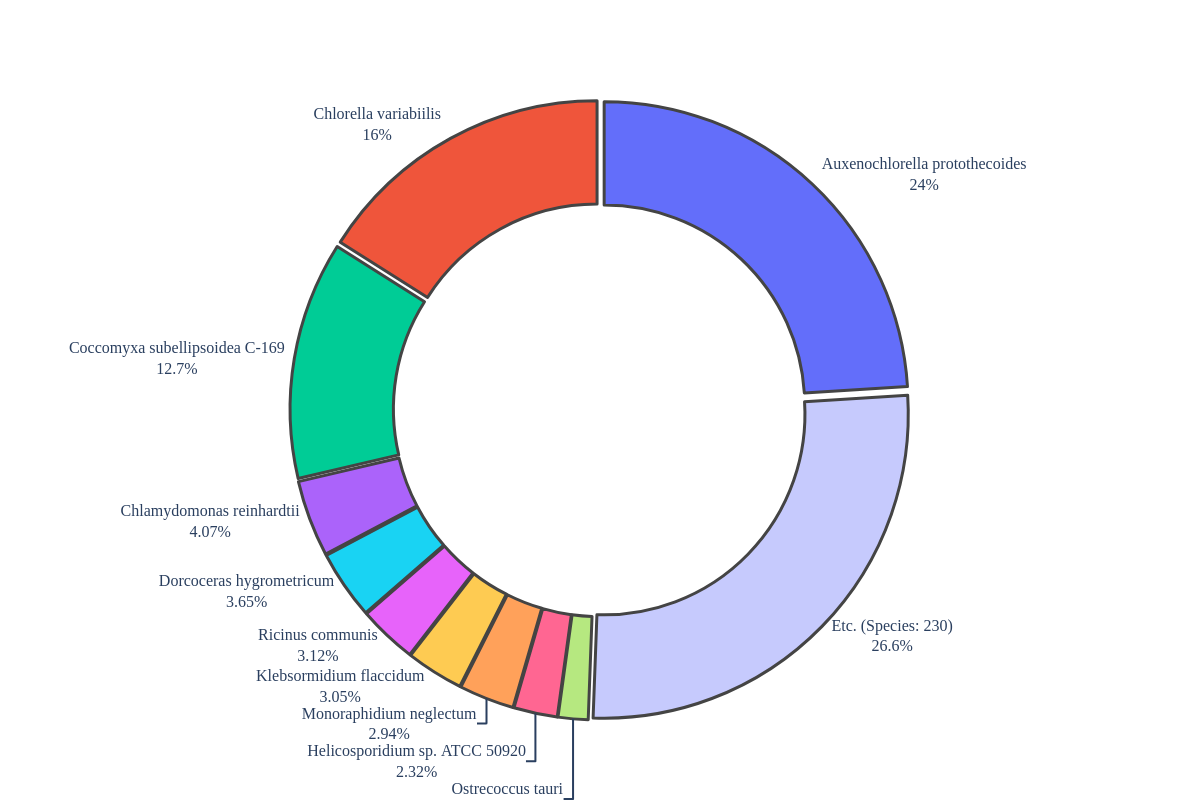


**Fig. S2** Gene ratio by species of genes used to annotate representative transcripts using NCBI NR.


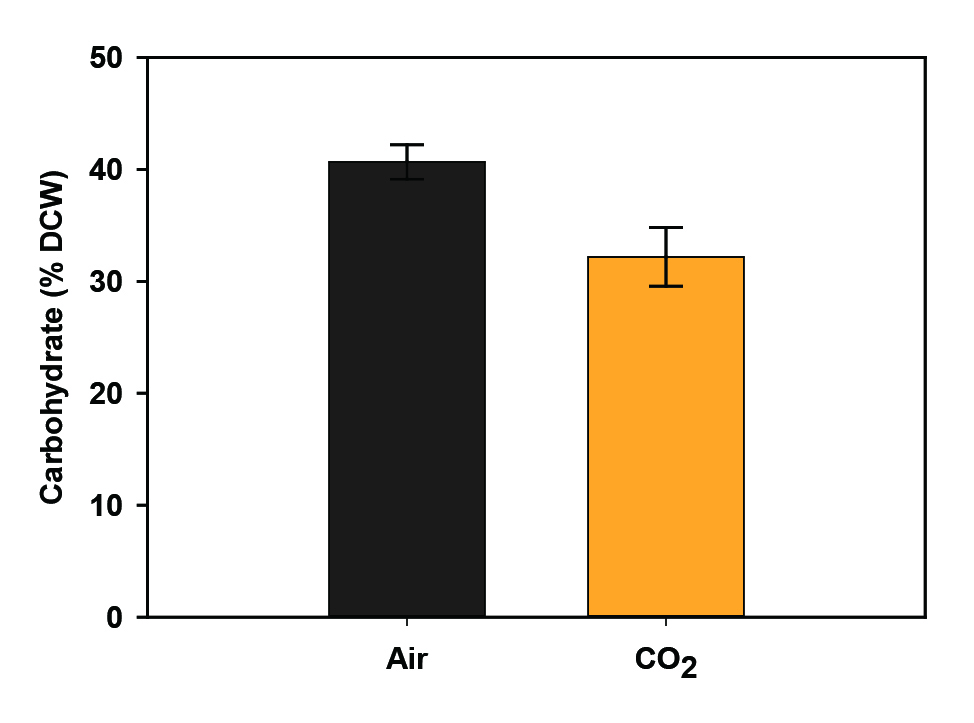


**Fig. S3** Carbohydrate content of cells cultivated under ambient air conditions and 10% CO_2_ conditions on day 7.


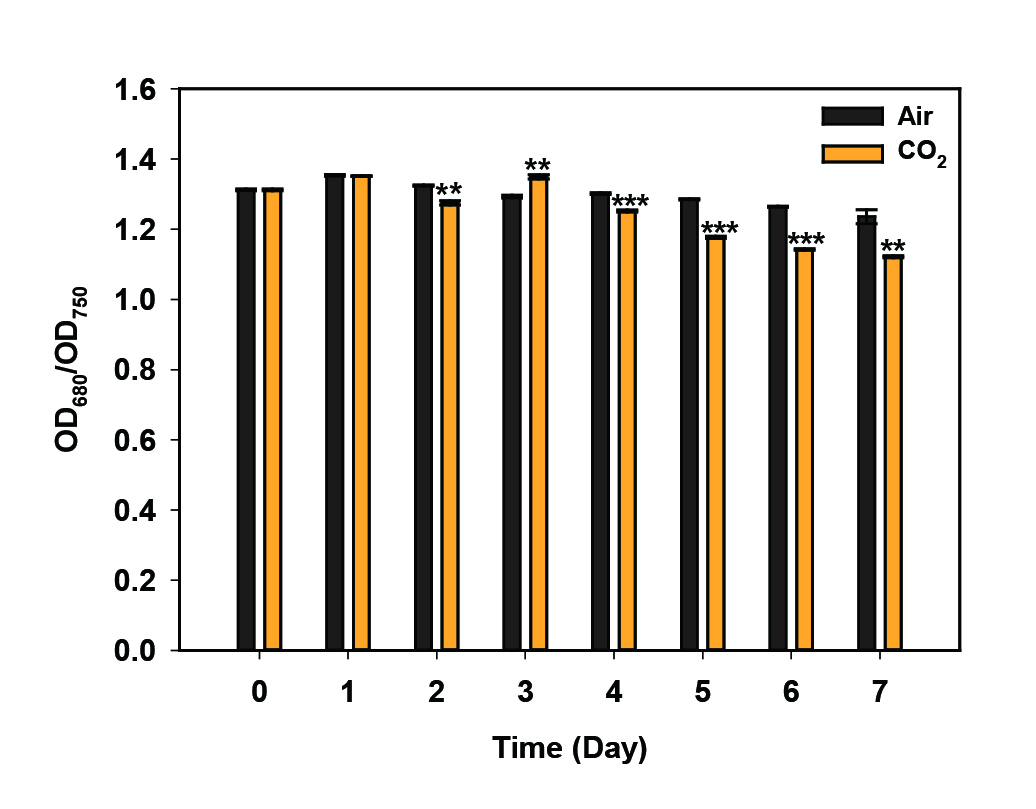


**Fig. S4** The changes in OD_680_/OD_750_ during the whole cultivation period. The ratio of OD_680_/OD_750_ is an approximate indicator of the photosynthetic efficiency and the physiological state of the cells in terms of their chlorophyll contents.


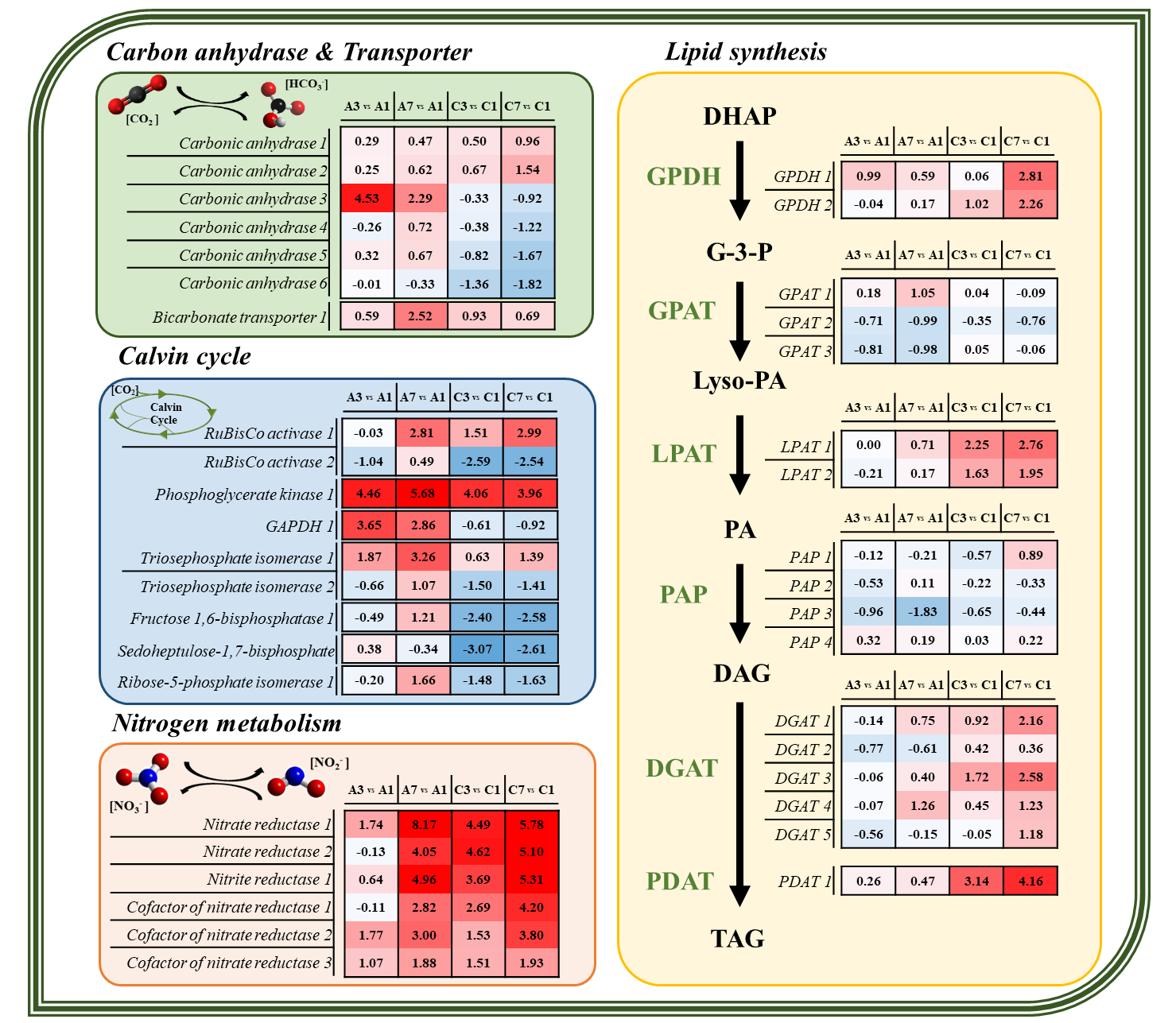


**Fig. S5** Relative expression levels (log_2_ fold change) of key enzymes participating in carbon fixation, Calvin cycle, nitrogen uptake, and lipid biosynthesis. Time-course comparison of cells cultivated in the same CO_2_ concentration (10% CO_2_ or ambient air) at different growth phase.


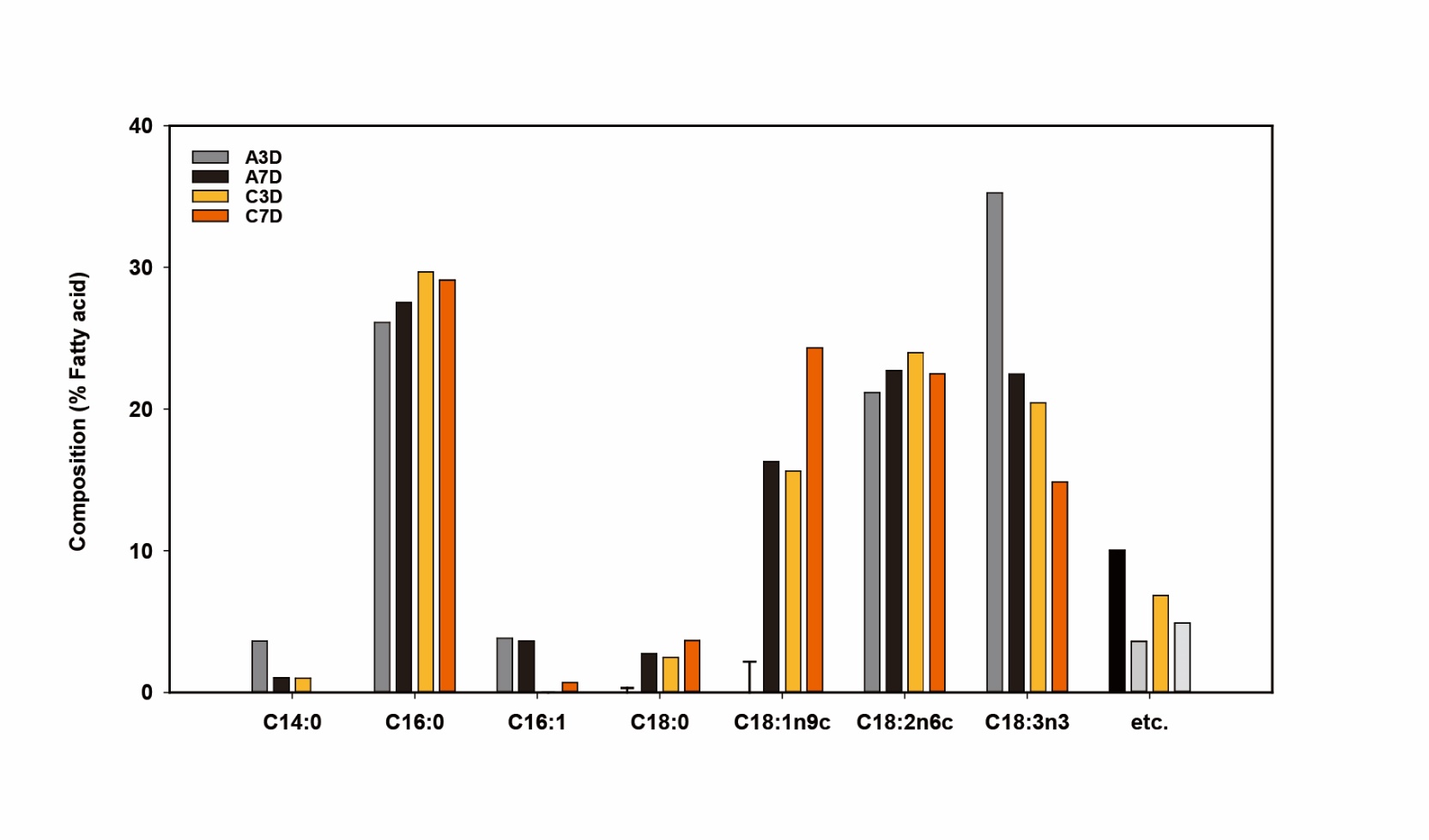


**Fig. S6** The profiles of fatty acid composition of cells under A3D, A7D, C3D, and C7D conditions. Error bars stand for the standard error calculated from three independent experimental data sets.


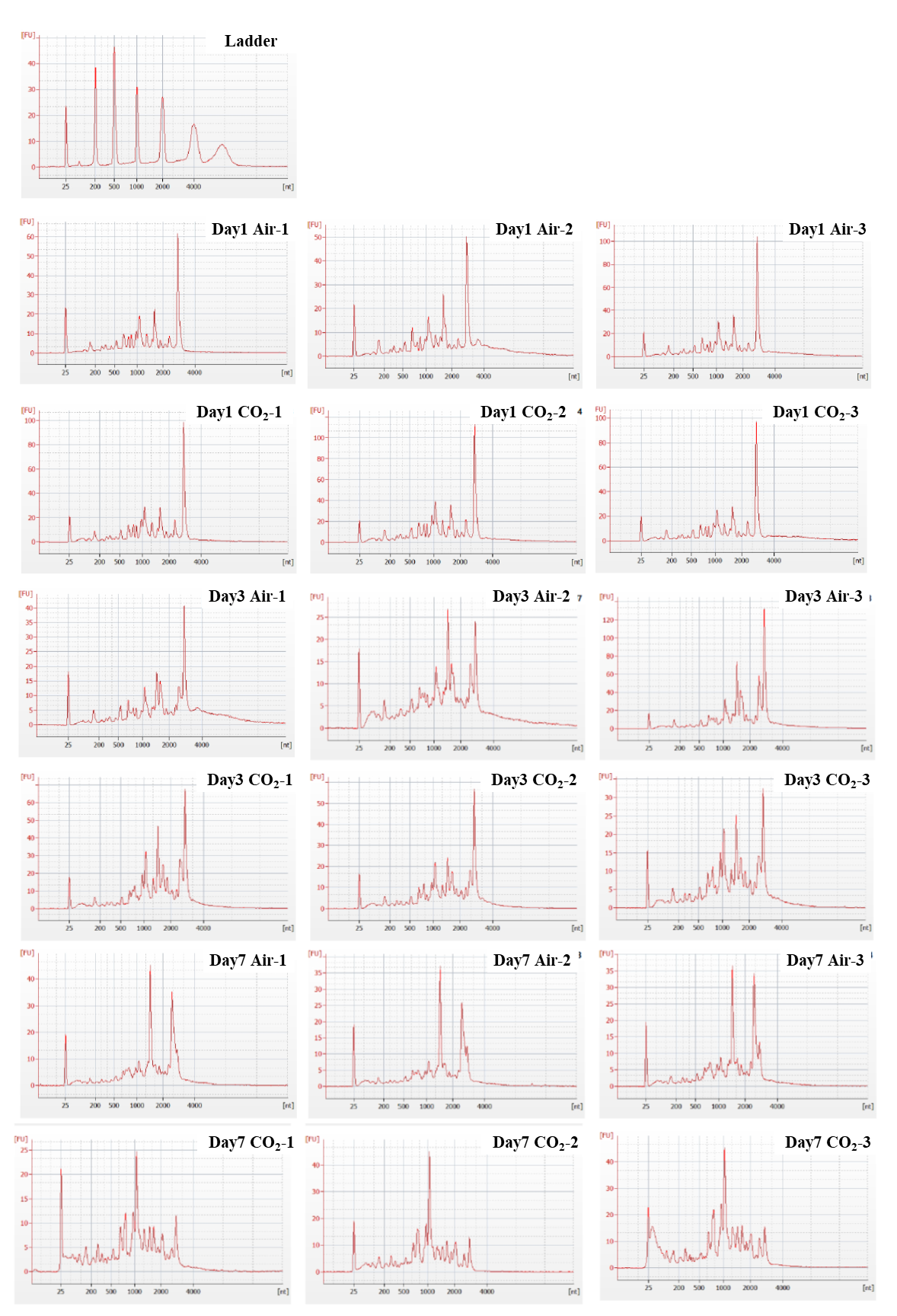


**Fig. S7** The RNA integrity electropherograms of each sample.


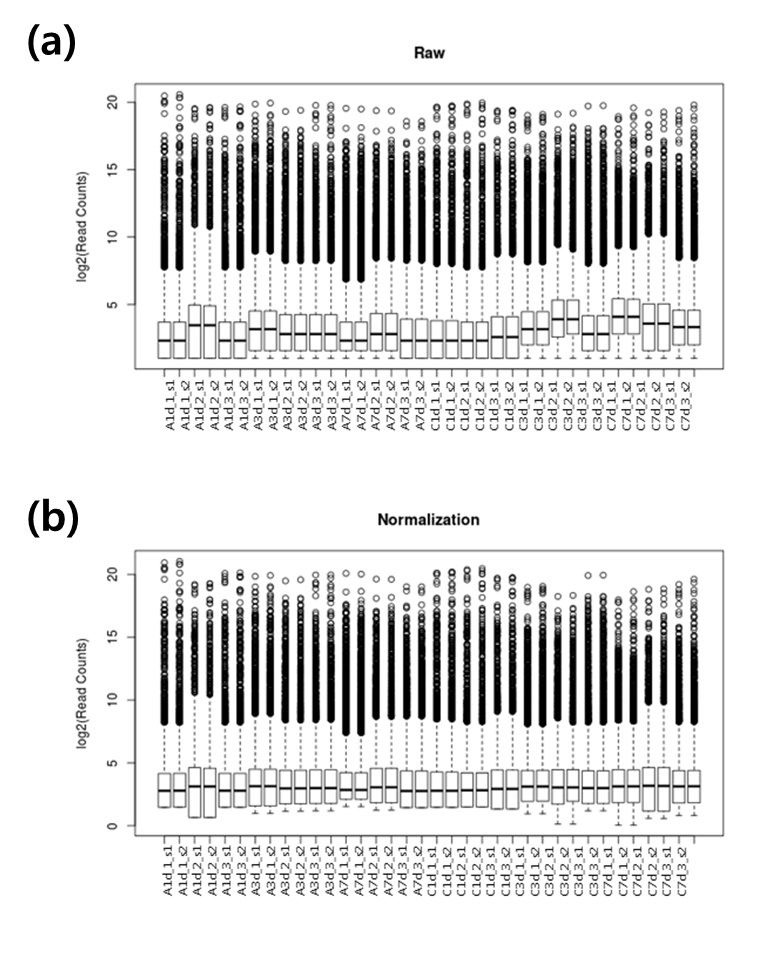


**Fig. S8** Boxplot of gene expression level before and after normalization. (a) Average expression level of the raw data from each sample (b) Average expression level of the normalized data from each sample


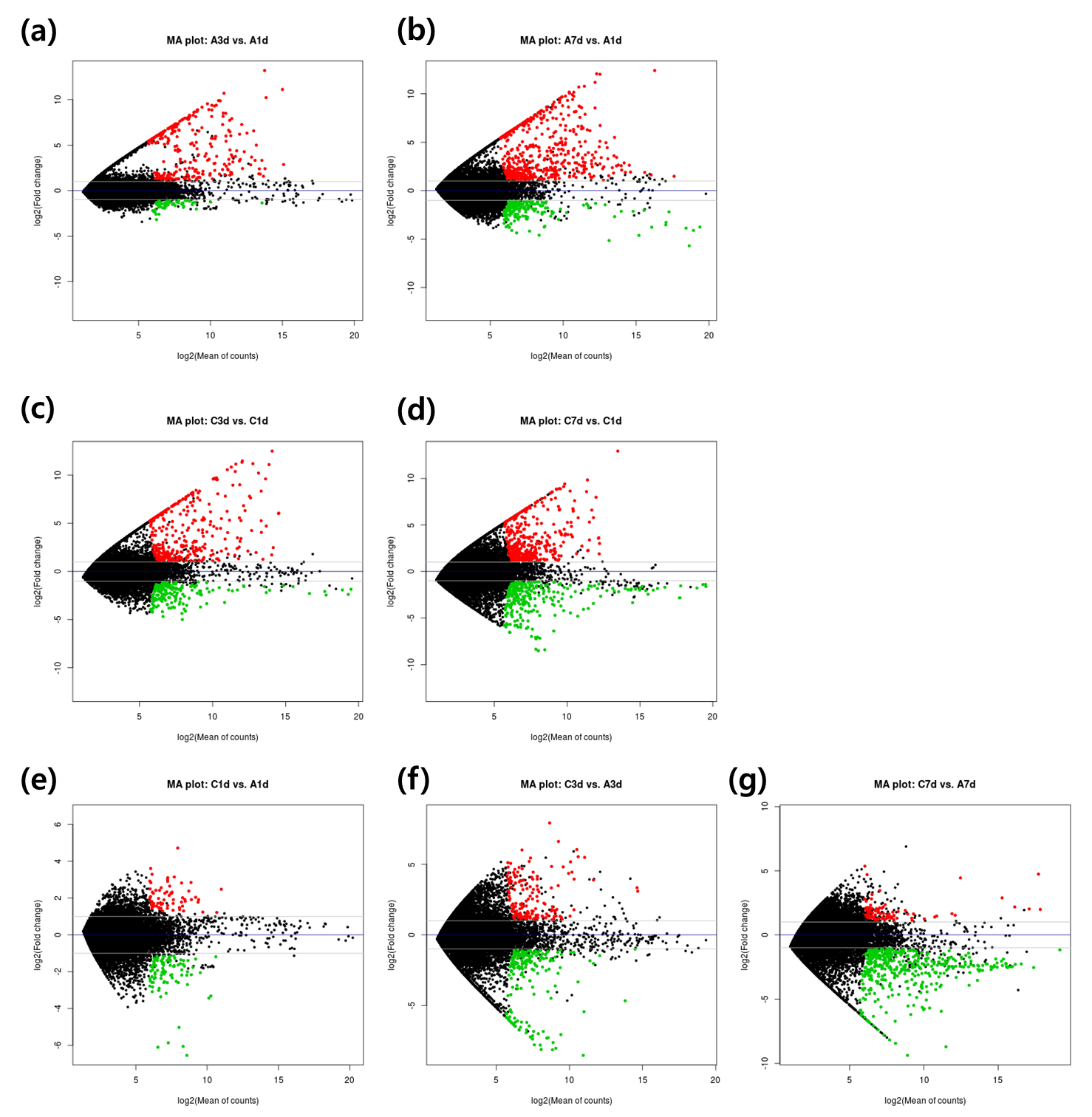


**Fig. S9** MA plot of DEGs in each condition. X-axis represents the average expression value of the control and sample on a log_2_ scale, and the Y-axis represents the fold change between the two samples, on a log_2_ scale.

**Supplementary Tables**

**Table S1.** Statistical results of annotated final unigenes.

| **Annotation database** | **Annotation no.** | **Annotation percentage (%)** |
| --- | --- | --- |
| NR viridiplantae | 9,178 | 69.4 |
| Phytozome | 7,032 | 53.17 |
| Uniprotkb viridiplantae | 8,591 | 67.68 |
| InterprotScan | 6,247 | 47.24 |
| KOG | 6,001 | 45.38 |
| KEGG | 2,968 | 22.44 |
| GO | 7,567 | 57.22 |
| **Total** | **10,246** | **77.47** |

| Target | Gene ID | Primer seq (5' - 3') |
| --- | --- | --- |
| *CA3* | Csorokiniana1SL013893t0001 | F: CCGTCAACCACTGGCAAAAG |
|  |  | R: TTTCGGCTTGGTATGCCCTT |
| *CA4* | Csorokiniana1SL004618t0001 | F: CCGTAATAGACCACGGAGGC |
|  |  | R: GGGCATGACCAAGAACCTGA |
| *CA5* | Csorokiniana1SL023565t0001 | F: CCGGATCACGACAAGGAGTG |
|  |  | R: ACGTTGAGCACGGAGATGTT |
| *NR1* | Csorokiniana1SL013132t0022 | F: CCGGACCATGTCTTCGTTGA |
|  |  | R: GACGACATCCTGCTTCGTGA |
| *CoNiR1* | Csorokiniana1SL010525t0003 | F: GCAGCGTGTCCAAACTGATG |
|  |  | R: GGGTGGATAAGATCCGCCTG |
| *TATA* | Csorokiniana1SL005261t0004 | F: ATAGCTGCAGAACATGGCGT |
|  |  | R: GCTAGCCTATGCGGTGTCTT |

**Table S2.** Primers used in this study for qRT-PCR.

| **Category** | **Name** | **Gene ID** | **Annotation** |
| --- | --- | --- | --- |
| **Carbonic**  **anhydrase** | *Carbonic anhydrase 1* | Csorokiniana1SL003404t0013 | Carbonic anhydrase 2 |
|  | *Carbonic anhydrase 2* | Csorokiniana1SL006190t0001 | Carbonic anhydrase 2 |
|  | *Carbonic anhydrase 3* | Csorokiniana1SL013893t0001 | Carbonic anhydrase 2 |
|  | *Carbonic anhydrase 4* | Csorokiniana1SL004618t0001 | Alpha carbonic anhydrase 4 |
|  | *Carbonic anhydrase 5* | Csorokiniana1SL023565t0001 | Alpha carbonic anhydrase 4 |
|  | *Carbonic anhydrase 6* | Csorokiniana1SL017994t0001 | Alpha carbonic anhydrase 7 |
| **Bicarbonate transporter** | *Bicarbonate transporter 1* | Csorokiniana1SL003509t0011 | Bicarbonate transporter, C-terminal |
| **Calvin Cycle** | *RuBisCo acitvase 1* | Csorokiniana1SL006892t0004 | RuBisCO activase-like protein |
|  | *RuBisCo acitvase 2* | Csorokiniana1SL009247t0003 | Rubisco activase |
|  | *Phosphoglycerate kinase 1* | Csorokiniana1SL025128t0002 | Phosphoglycerate kinase 1 |
|  | *GAPDH 1* | Csorokiniana1SL025688t0001 | Glyceraldehyde-3-phosphate dehydrogenase B subunit |
|  | *Triosephosphate isomerase 1*  *Triosephosphate isomerase 2* | Csorokiniana1SL025138t0001 | Triosephosphate isomerase  Triosephosphate isomerase |
|  |  | Csorokiniana1SL009514t0001 |  |
|  | *Fructose 1,6-bisphosphatase 1* | Csorokiniana1SL012635t0001 | Fructose-1,6-bisphosphatase |
|  | *Sedoheptulose-1,7-bisphosphate* | Csorokiniana1SL012385t0001 | Sedoheptulose-bisphosphatase |
|  | *Ribose-5-phosphate isomerase 1* | Csorokiniana1SL005037t0001 | Ribose 5-phosphate isomerase, type A protein |
| **Nitrogen**  **metabolism** | *Nitrate reductase 1* | Csorokiniana1SL013132t0022 | Nitrate reductase 1 |
|  | *Nitrate reductase 2* | Csorokiniana1SL015976t0001 | Nitrate reductase 1 |
|  | *Nitrite reductase 1* | Csorokiniana1SL000622t0009 | Nitrite reductase 1 |
|  | *Cofactor of nitrate reductase 1* | Csorokiniana1SL010525t0003 | Cofactor of nitrate reductase and xanthine dehydrogenase 2 |
|  | *Cofactor of nitrate reductase 2* | Csorokiniana1SL026782t0001 | Cofactor of nitrate reductase and xanthine dehydrogenase 3 |
|  | *Cofactor of nitrate reductase 3* | Csorokiniana1SL009238t0008 | Co-factor for nitrate, reductase and xanthine dehydrogenase 7 |
| **Lipid synthesis** | *GPDH 1* | Csorokiniana1SL005537t0009 | NAD-dependent glycerol-3-phosphate dehydrogenase family protein |
|  | *GPDH 2* | Csorokiniana1SL008991t0002 | NAD-dependent glycerol-3-phosphate dehydrogenase family protein |
|  | *GPAT 1* | Csorokiniana1SL022793t0002 | Glycerol-3-phosphate acyltransferase, chloroplastic |
|  | *GPAT 2* | Csorokiniana1SL004054t0001 | Glycerol-3-phosphate 1-acyltransferase |
|  | *GPAT 3* | Csorokiniana1SL003657t0015 | Glycerol-3-phosphate acyltransferase 3 |
|  | *LPAT 1* | Csorokiniana1SL001137t0013 | Acyl transferase/acyl hydrolase/lysophospholipase |
|  | *LAPT 2* | Csorokiniana1SL005237t0010 | Acyl transferase/acyl hydrolase/lysophospholipase |
|  | *PAP 1* | Csorokiniana1SL008676t0001 | Phosphatidic acid phosphatase type 2/haloperoxidase |
|  | *PAP2* | Csorokiniana1SL008141t0002 | Phosphatidic acid phosphatase type 2/haloperoxidase |
|  | *PAP 3* | Csorokiniana1SL001005t0007 | Phosphatidic acid phosphatase type 2/haloperoxidase |
|  | *PAP 4* | Csorokiniana1SL002316t0001 | Lysophosphatidic acid phosphatase type 6 |
|  | *DGAT 1* | Csorokiniana1SL002323t0008 | Diacylglycerol O-acyltransferase 1 |
|  | *DGAT 2* | Csorokiniana1SL005875t0001 | Diacylglycerol acyltransferase |
|  | *DGAT 3* | Csorokiniana1SL001950t0010 | Diacylglycerol acyltransferase family |
|  | *DGAT 4* | Csorokiniana1SL007263t0011 | Diacylglycerol acyltransferase family |
|  | *DGAT 5* | Csorokiniana1SL020759t0003 | Diacylglycerol acyltransferase family |
|  | *PDAT 1* | Csorokiniana1SL007740t0001 | Phospholipid:diacylglycerol acyltransferase |
|  | *bHLH* | Csorokiniana1SL018303t0001 | Basic region/leucine zipper transcription factor 68 |
|  | *MYB* | Csorokiniana1SL021050t0001 | MYB family transcription factor |
|  | *bZIP1* | Csorokiniana1SL008762t0002 | Basic-leucine zipper (bZIP) transcription factor family protein |
|  | *bZIP2* | Csorokiniana1SL004188t0003 | Basic helix-loop-helix (bHLH) |
|  | *ACC1* | Csorokiniana1SL006434t0001 | Acetyl-CoA carboxylase 1 |
|  | *β-oxidase 1* | Csorokiniana1SL001593t0002 | Putative glyoxysomal fatty acid beta-oxidation multifunctional protein |
|  | *β-oxidase 2* | Csorokiniana1SL008557t0003 | Glyoxysomal fatty acid beta-oxidation multifunctional protein MFP-a |
|  | *Δ12 FAD 1* | Csorokiniana1SL002731t0007 | Delta-12 fatty acid desaturase |
|  | *Δ12 FAD 2* | Csorokiniana1SL018441t0003 | Plastid delta12 fatty acid desaturase |
|  | *Δ12 FAD 3* | Csorokiniana1SL002731t0007 | Delta-12 fatty acid desaturase |
|  | *Δ12 FAD 4* | Csorokiniana1SL018441t0003 | Plastid delta12 fatty acid desaturase |
|  | *FAD 1* | Csorokiniana1SL007969t0009 | Sphingolipid delta(4)-desaturase/C4-hydroxylase DES2, Fatty acid desaturase domain |
|  | *FAD 2* | Csorokiniana1SL010241t0005 | Acyl-carrier-protein desaturase, Fatty acid desaturase, type 2 |
|  | *FAD 3* | Csorokiniana1SL011129t0007 | Fatty acid desaturase, type 1 |
|  | *FAD 4* | Csorokiniana1SL025173t0001 | Acyl-[acyl-carrier-protein] desaturase 6, chloroplastic, Fatty acid desaturase domain |
|  | *FAD 5* | Csorokiniana1SL026756t0001 | Acyl-CoA desaturase, Fatty acid desaturase domain |
|  | *FAD 6* | Csorokiniana1SL002773t0008 | Fatty acid desaturase domain |
| **Etc.** | *ADH* | Csorokiniana1SL006387t0009 | Aldehyde dehydrogenase 10A9 |
|  | *FeH* | Csorokiniana1SL002971t0015 | FeFe-hydrogenase assembly protein |
|  | *METE* | Csorokiniana1SL000035t0006 | Cobalamin-independent methionine synthase |
|  | *SS* | Csorokiniana1SL003579t0005 | Starch synthase 4 |

**Table S3.** Gene IDs of key enzymes described in Fig. 4 ,5, and 6
